# The 5′-terminal stem–loop RNA element of SARS-CoV-2 features highly dynamic structural elements that are sensitive to differences in cellular pH

**DOI:** 10.1093/nar/gkae477

**Published:** 2024-06-06

**Authors:** Sabrina Toews, Anna Wacker, Edgar M Faison, Elke Duchardt-Ferner, Christian Richter, Daniel Mathieu, Sandro Bottaro, Qi Zhang, Harald Schwalbe

**Affiliations:** Institute of Organic Chemistry and Chemical Biology, Johann Wolfgang Goethe-University Frankfurt, Frankfurt/Main, Hesse 60438, Germany; Center for Biomolecular Magnetic Resonance (BMRZ), Johann Wolfgang Goethe-University Frankfurt, Frankfurt/Main, Hesse 60438, Germany; Institute of Organic Chemistry and Chemical Biology, Johann Wolfgang Goethe-University Frankfurt, Frankfurt/Main, Hesse 60438, Germany; Center for Biomolecular Magnetic Resonance (BMRZ), Johann Wolfgang Goethe-University Frankfurt, Frankfurt/Main, Hesse 60438, Germany; Department of Biochemistry and Biophysics, University of North Carolina at Chapel Hill, Chapel Hill, NC27599, USA; Center for Biomolecular Magnetic Resonance (BMRZ), Johann Wolfgang Goethe-University Frankfurt, Frankfurt/Main, Hesse 60438, Germany; Institute of Molecular Biosciences, Johann Wolfgang Goethe-University Frankfurt, Frankfurt/Main, Hesse 60438, Germany; Institute of Organic Chemistry and Chemical Biology, Johann Wolfgang Goethe-University Frankfurt, Frankfurt/Main, Hesse 60438, Germany; Center for Biomolecular Magnetic Resonance (BMRZ), Johann Wolfgang Goethe-University Frankfurt, Frankfurt/Main, Hesse 60438, Germany; Bruker BioSpin GmbH, Ettlingen, Baden-Württemberg 76275, Germany; Linderstrøm-Lang Centre for Protein Science, Department of Biology, University of Copenhagen, Copenhagen 2200, Denmark; Department of Biochemistry and Biophysics, University of North Carolina at Chapel Hill, Chapel Hill, NC27599, USA; Institute of Organic Chemistry and Chemical Biology, Johann Wolfgang Goethe-University Frankfurt, Frankfurt/Main, Hesse 60438, Germany; Center for Biomolecular Magnetic Resonance (BMRZ), Johann Wolfgang Goethe-University Frankfurt, Frankfurt/Main, Hesse 60438, Germany

## Abstract

We present the nuclear magnetic resonance spectroscopy (NMR) solution structure of the 5′-terminal stem loop 5_SL1 (SL1) of the SARS-CoV-2 genome. SL1 contains two A-form helical elements and two regions with non-canonical structure, namely an apical pyrimidine-rich loop and an asymmetric internal loop with one and two nucleotides at the 5′- and 3′-terminal part of the sequence, respectively. The conformational ensemble representing the averaged solution structure of SL1 was validated using NMR residual dipolar coupling (RDC) and small-angle X-ray scattering (SAXS) data. We show that the internal loop is the major binding site for fragments of low molecular weight. This internal loop of SL1 can be stabilized by an A12–C28 interaction that promotes the transient formation of an A^+^•C base pair. As a consequence, the p*K*_a_ of the internal loop adenosine A12 is shifted to 5.8, compared to a p*K*_a_ of 3.63 of free adenosine. Furthermore, applying a recently developed pH-differential mutational profiling (PD-MaP) approach, we not only recapitulated our NMR findings of SL1 but also unveiled multiple sites potentially sensitive to pH across the 5′-UTR of SARS-CoV-2.

## Graphical Abstract

**Graphical Abstract:**
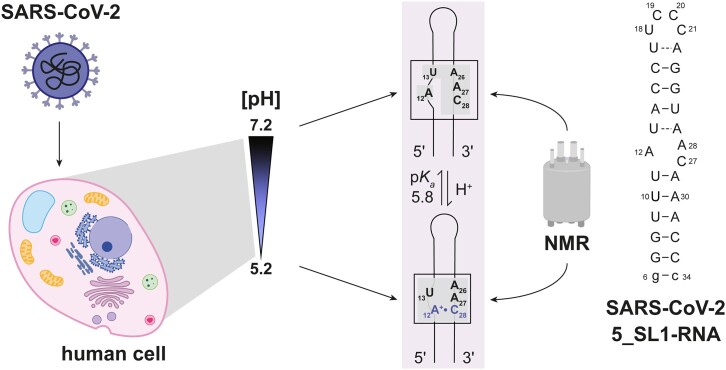

## Introduction

**Figure 1.**
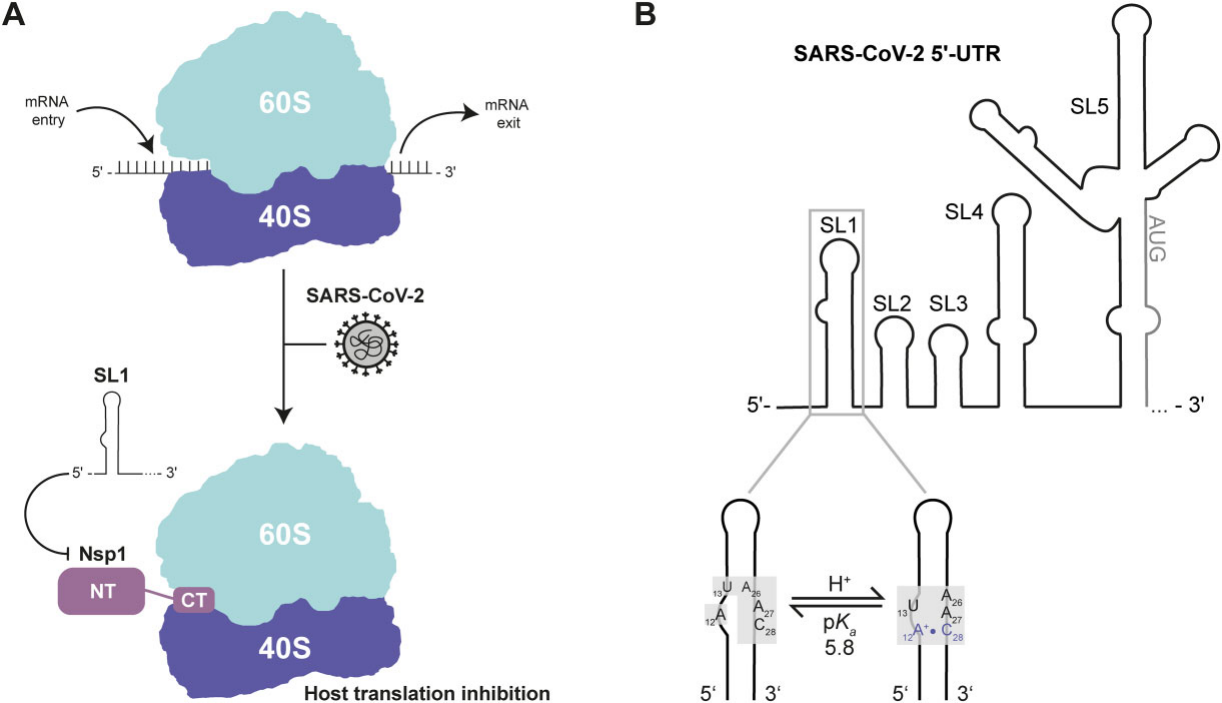
Biological context of SL1 and the secondary structure of SL1 highlighting its protonation site. (**A**) Host mRNA translation inhibition induced by SARS-CoV-2. Nsp1 blocks the mRNA entry channel of the 40S ribosomal subunit. Proposed models indicate that SL1 might be needed to escape the blocked mRNA channel via an interaction with Nsp1 and to allow viral translation in infected host cells. (**B**) Genomic context of SL1 highlighting the pH-induced rearrangement of the internal loop.

The RNA genome of the severe acute respiratory syndrome coronavirus 2 (SARS-CoV-2) contains multiple regulatory RNA elements, especially within its untranslated regions (UTR) at the genome termini. Stem–loop 1 (SL1) is located at the 5′-terminus of the viral genome, comprising nucleotides 7–33. It is strongly conserved in the *Coronaviridae* family and is supposed to act as a universal replication signal (1). The architecture and dynamics of this RNA element have been comprehensively characterized by NMR spectroscopy in the model Betacoronavirus (βCoV) *murine hepatitis virus* (MHV), and differential stability of its two helical parts were shown to be essential for genome cyclization (2,3). In human βCoVs, a key function of SL1 is to bypass the occlusion of the ribosomal mRNA entry site by the viral non-structural protein 1 (Nsp1), allowing viral mRNA to be translated (4–7). This function of SL1 is sensitive to (i) the sequence of the nucleotides 15–21 (8,9) that are involved in positioning the SL1 stem–loop with respect to the 5′-end of the mRNA (8), (ii) key residues within the N-terminal domain of the viral protein Nsp1 (10), (iii) the Nsp1 linker length that connects the ribosome-binding C-terminal domain and RNA-binding N-terminal domain (4) and (iv) the activity of the eukaryotic initiation factor 4A (eIF4A) (11). A recent study showed that the mechanism of Nsp1 evoking the inhibition of host-translation within coronaviruses is genera-dependent and the shutdown via high-affinity binding to the mRNA entry site of the host-ribosome is specific for βCoVs (Figure 1A) (12). Together, these results consistently highlight the functional importance of SL1 and point out its potential as a target for antiviral therapies against SARS-CоV-2. Indeed, antisense oligonucleotides (ASOs) targeting the stem–loop structure of SL1 have demonstrated promising antiviral efficacy (9,13).

Our lab investigated the druggability of the viral RNA genome via nuclear magnetic resonance spectroscopy (NMR) with low molecular-weight compounds. These experiments identified particular candidates that target the pertinent viral RNA (14). To delineate the binding site of SL1 and to set the structural basis for subsequent research on the RNA’s role in the viral life cycle of SARS-CoV-2, we here determined the NMR- and small angle scattering (SAXS)-derived structural ensemble of SARS-CoV-2 SL1. In our previous work, we validated the strategy to investigate the isolated RNA stem–loops from the 5′-UTR, as there is no evidence for higher-order structure formation within this genomic region (15). In general, there are no experimental NMR-determined RNA structures available for the SARS-CoV-2 5′-UTR, except for stem–loop 2 (16) and stem–loop 4 (17).

In our structure calculations, we observed an interaction between A12 and C28 in the internal loop in a significant proportion of conformational states (Figure 1B). This observation prompted us to speculate that the spatial proximity and near coplanar orientation of those nucleobases could shift the p*K*_a_ of the involved adenosine, promoting the formation of an A^+^•C wobble base pair. We thus characterized the pH-dependence of structure, dynamics and global stability of SL1 for pH values between 5.2 and 7.2. pH-sensitive experiments revealed protonation of A12 and show that this transient N1-protonation already occurs at pH 6.2, reflected in broadening of NMR signals beyond detectability. Interestingly, reconciling NMR and circular dichroism (CD) spectroscopy data reveals that A12 N1 exhibits a p*K*_a_ of 5.8 and that A12 protonation leads to a global destabilization of the SL1 stem–loop despite transient A^+^•C-wobble base pair formation. According to its p*K*_a_, the A^+^•C wobble base pair is populated to 6% at neutral pH, leading to conformational heterogeneity of the internal loop conformation. This pH-dependent modulation of the internal loop conformation is important, as we show here that it is the target site for binding of fragments of low molecular weight.

pH-dependent structural dynamics are also present in other RNA elements of the SARS-CoV-2 5′-UTR including the 5_SL4 stem loop structure showing elevated p*K*_a_ values within non-canonical structure motifs (17). Indeed, the pH-differential mutational profiling (PD-MaP) analysis (18) of the 5′-UTR at a pH range covering the physiologically relevance (pH 5.0–8.0) reported here confirms the existence of p*K*_a_-shifted sites in all RNA stem–loops in the 5′-UTR of SARS-CoV-2, which emphasizes the importance to examine pH sensitivity closely. In line, the significance of examining the impact of pH on RNA structure and function has become apparent recently (19–24).

The dynamic NMR structural ensemble, altered at different pH, of the stem–loop SL1 will support further studies to elucidate the exact molecular mechanism of the proposed SL1-Nsp1 interaction centrepiece. In the context of targeting elements of the RNA genome by low molecular weight compounds (14), we show that the dynamic internal loop structure can be targeted by fragment ligands.

## Materials and methods

### RNA synthesis

RNA samples were prepared as described in Wacker and Weigand *et al.* (15). Additionally, unlabeled SL1 RNA was purchases from Horizon Discovery and was used for selected NMR and CD measurements (as indicated). Briefly, if synthesized in house, previously amplified and linearized double-stranded template DNA was incubated for 6 h at 37°C together with the bacteriophage-derived RNA-polymerase T7 along with co-factors and substrates (nucleoside triphosphates, Mg^2+^, dithiothreitol, spermidine) buffered in 0.2 M Tris–HCl pH 8.0 (25,26). *In vitro* transcribed RNA was purified via polyacrylamide gel purification under denaturing conditions and further processed with reverse-phase (RP) high-performance liquid chromatography (HPLC). Final RNA samples were buffer exchanged to 25 mM potassium phosphate (KP_i_) and 50 mM potassium chloride (KCl) using 3-kDa molecular weight cut-off (MWCO) VivaSpin filtration units (Sartorius). For all NMR experiments dedicated to structure calculation, the pH was 6.2. Variable pH values were achieved by adjusting the KP_i_ buffer composition of mono- and dibasic KP_i_. pH values below 6.0 were achieved by adding HCl.

### NMR experiments

NMR experiments for the assignment of the SL1 protons were carried out as previously described (27). For structure calculations, additional nuclear Overhauser enhancement and exchange spectroscopy (NOESY) spectra with homogeneous excitation profiles for nuclear Overhauser effect (NOE)-derived distance restraints were measured each for the exchangeable protons (in 5% D_2_O; pH 6.2, 283 K; 80 ms NOESY mixing time) and for non-exchangeable protons (in 99.95% D_2_O; pH 6.2, 298 K; 150, 200 and 300 ms NOESY mixing times), respectively. To collect sufficient NOE intensities from resolved NOESY cross-peaks, we recorded ^13^C{F2}-edited and ^13^C{F2}-filtered NOESY spectra on a selectively {A,C}-^13^C,^15^N-labeled RNA (28). NOESY peak picking and peak list generation were performed with NMRFAM-Sparky (29).

{^1^H}–^13^C heteronucler NOEs (hetNOEs) were measured at pH 6.2 and pH 5.2 using constant-time chemical shift evolution in the ^13^C dimension, with and without NOE build-up in an interleaved manner and with temperature compensation. The inter-scan delay was set to 5 s and the number of scans was set to 64 to achieve a signal-to-noise ratio >50 for both NOE- and noNOE spectra. hetNOE values were extracted from the signal intensity ratio of NOE and noNOE spectra using Dynamics Center (Bruker).

pH titrations were carried out by rebuffering the RNA sample in KP_i_ buffer of the appropriate pH and subsequent determination of the final sample pH using the internal ^31^P NMR signal. Calibration for the pH-dependent ^31^P shift of the P_i_ buffer signal was performed at 500 and 700 MHz using sodium trimethylsilylpropanesulfonate (DSS) as internal reference. The p*K*_a_ of A12 was estimated by fitting its C2 chemical shift to the following equation in Matlab:


(1) \begin{eqnarray*}\Delta C2 = \ \Delta C{{2}_{N1H}}\ + \ \left( {\frac{{\Delta C{{2}_{N1 - }}\Delta C{{2}_{N1H + }}}}{{1 + {{{10}}^{p{{K}_a}_{ - pH}}}}}} \right)\end{eqnarray*}

For a given pH, the population of RNAs protonated at A12N1 was calculated from the Henderson–Hasselbalch relationship:


(2) \begin{eqnarray*}{\rm pH}\ = \ {\rm p}K_{\rm a}\ + \ {\rm log}\left( {\frac{{\left[ {{\rm A} - } \right]}}{{\left[ {{\rm HA}} \right]}}} \right)\end{eqnarray*}

Super-cooled NMR experiment at temperatures below 273 K were carried out in eleven glass capillaries with a diameter of 1 mm placed into a conventional 5 mm tube. This method allowed further cooling of the RNA sample below 273 K due to increased surface (30,31).

### RDC determination

CH and NH RDC values were extracted from the difference in undecoupled ^1^H,^15^N correlation experiments in the direct dimension in Pf1 filamentous bacteriophage-aligned versus non-aligned SL1 RNA samples. Partial alignment of SL1 was achieved by the addition of 10 mg/mL Pf1 phages, yielding a deuterium splitting of 11 Hz at 600 MHz. Peak picking and RDC determination were performed in Bruker Topspin 4.1.4 software. RDC values were used to iteratively estimate rhombicity (final value: 2.7) and anisotropy (final value: 19) of the alignment tensor by using preliminary 3D coordinates generated from structure calculations without the incorporation of RDCs in PALES (32).

### Ligand-based titrations via NMR for the determination of binding affinities

Binding affinity of the fragment C11 was determined via NMR as published in Sreeramulu and Richter *et al.* (33). The concentrations of the investigated RNAs were kept constant whilst the concentration of the fragment was steadily increased. For this, eight individual samples were prepared in 1.7 mm NMR tubes with a total volume of 40 μl including 100 μM of RNA and 0 to 250 μM of fragment C11. Samples were buffered in 25 mM KP_i_ (pH 6.2), 50 mM KCl and 5% deuterated dimethyl sulfoxide (DMSO-d_6_) as lock solvent and reference. Final 1D-^1^H NMR experiments were conducted at 298 K and 600 MHz using excitation sculpting for solvent suppression. Changes in chemical shift perturbations (CSPs) were analysed and further processed for the determination of binding affinities with a non-linear fit equation assuming one site-specific binding (Eq. (3)). In this equation, *X* refers to the used RNA concentrations in μM and *B*_max_ to the maximum number of binding sites.


(3) \begin{eqnarray*}{\rm CSP}\ \left[ {{\rm Hz}} \right] = \frac{{{{B}_{{\rm max}\ }}x}}{{{{K}_{\rm D}}^{{\rm est}}\ + \ x}}\end{eqnarray*}

We provide estimated dissociation constants (*K*_D_^est^) values as concentration dependent CSPs often do not achieve saturation.

### Binding site mapping via NMR

Chemical shift changes induced on NMR signals of SL1 upon addition of fragment C11 were used to delineate the interaction site of the target RNA. NOEs between ligand C11 and RNA were not observed, presumably due to weak binding affinity.

To validate shifted peaks properly, two different samples were prepared using the NMR buffer provided with 5% DMSO-d_6_. SL1 was measured alone with a final concentration of 150 μM and further in the presence of 1 mM C11. Excess of ligand was utilized to ensure binding to SL1. Both, the RNA and the fragment were unlabelled. Assigned RNA peaks of interest were analysed with 1D- and 2D-NMR experiments at 298 K and 600 MHz.

### 2-Aminopurine *in vitro* assay for the determination of binding affinities

As an alternative method for the determination of binding affinities, an *in vitro* assay based on the 2-aminopurine (2AP) labeling of SL1 was established. SL1 was modified at position 27 by exchanging the adenine residue by 2AP. RNA was ordered from Horizon Discovery and rebuffered to NMR buffer with a pH of 7.2. The intrinsic fluorescence of 2AP was measured in the presence of increasing fragment concentrations whilst the RNA concentration was kept constant. Samples were prepared separately with an individual blank sample containing the compound alone in the respective concentration as the sample with SL1 and C11 to exclude any intrinsic fluorescence of the compound itself. Samples were transferred to a black flat-bottom 384-well plate with a total volume of 30 μl in triplicates and incubated for 30 min at 25°C. Fluorescence measurements were carried out using a Tecan Spark® microplate reader (Tecan, Männedorf, Switzerland). 2AP fluorescence was excited at wavelength of 308 nm and emitted light was detected at 372 nm with a bandwidth of 10 nm at 25°C. Data was evaluated using GrapPad Prism 5.0 and fitted with Equation (3) for the determination of binding affinities.

### Structure calculations

Structure calculations were performed with ARIA 1.2 (34) using CNS 1.1 (35) and the adapted force field dna-rna-allatom-hj (36). NOE restraints from five different 2D-NOESY spectra with mixing times of 80, 150 (2×), 200 and 300 ms were incorporated, yielding a total number of 372 unambiguous NOE restraints. Spin diffusion was corrected for within the ARIA routine. From the spectra measured at 283 K, only NOEs stemming from those parts of the RNA that showed negligible temperature-dependent chemical shift perturbations (CSPs) were used in the structure calculations. We further restrained H-bonds for intra-base pair ^*2*h^*J*_NN_ couplings >4 Hz (base pairs G6–C34 to U11–A29 and U13–A26 to U17–A22) and applied base co-planarities for these base pairs. For the final iteration of the structure calculation, aromatic CH RDCs for adenosine residues were incorporated. NH RDCs were left out for cross-validation of the final structures. Standard A-form helix backbone torsion angles for nucleotides G8 to U11; U13 to U17; A22 to A26 and A29 to C33 were applied. Further, *α* and *ζ* torsion angles were restrained to A-form for all residues showing canonical ^31^P chemical shifts (37,38). Nucleotides lacking H1′, H2′ cross peaks in homonuclear TOCSY experiments were restrained to ribose 3‘-*endo* conformation, in agreement with canonical coordinates derived from ribose ^13^C chemical shifts (39). The glycosidic torsion angles *χ* for nucleotides showing weak-to-moderate H1′–H8 (purine) or H1′–H6 (pyrimidine) NOE intensities were set to *anti*-conformation. A total of eight iterations were calculated for each ensemble, with 100 structures per iteration. The 20 lowest-energy structures were used for each following iteration. The final iteration was performed with a total of 200 structures where the 20 lowest-energy structures were subjected to water refinement. Ten out of the 20 lowest-energy structures were finally selected according to their correlation of back-calculated RDCs with the total set of 18 experimental RDCs. The correlation score was determined with PALES using the bestFit option (32). Clash scores and structure geometries were analyzed with MolProbity and w3DNA (37,40). Finally, 3D coordinates of the 10-structure bundle were converted to mmCIF format using the wwPDB validation system webservers (https://validate.wwpdb.org).

### SAXS experiments

RNA samples for SAXS were prepared as described previously (15) at a concentration range of 1.5–3.5 mg/ml (corresponding to 160–370 μM) and in SAXS buffer (50 mM BisTris, 25 mM NaCl, pH 6.2). Synchrotron Small Angle X-ray scattering (SAXS) data were collected on the EMBL P12 beamline at the PETRA III storage ring (DESY, Hamburg, Germany) using a Pilatus 2M detector at a sample-detector distance of 3.1 m and a wavelength of *λ* = 0.124 nm. Data were measured at 298 K at continuous flow with a total exposure time of 3.135 s (33 × 95 ms frames). Automated processing included normalization of scattering data to the intensity of the transmitted beam and radial averaging as well as subtraction of the buffer scattering data. SAXS curves were plotted as *I*(*s*) versus *s*, where *s = 4π*sin *θ/λ*, and 2*θ* is the scattering angle. The particle distance distribution function *P*(*r*) plots were calculated using GNOM (41). SAXS curve fits to experimental structures were calculated using CRYSOL via the ATSAS online package (42). The distance distribution function *P*(*r*) for a sub-range of SAXS data (0.5 < s < 4.5 nm^−1^) was used for *ab initio* model building using DAMMIN (ATSAS online) (43).

### Circular dichroism spectroscopy and melting curve analysis

Circular dichroism (CD) experiments were performed using a Jasco J-810 spectropolarimeter equipped with a Peltier temperature controller (JASCO Applied Sciences GmbH). CD spectra were acquired over a wavelength range of 200–320 nm at various temperatures, using a 1 mm quartz cuvette (Hellma 110-QS, Hellma GmbH) with a final RNA concentration of 10 μM in a 200 μl sample volume. The RNA samples were measured in the NMR buffer with different pH values in a temperature range of 5–95°C. Each spectrum was averaged from five accumulations, recorded at a scanning speed of 100 nm/min with a data interval of 0.5 nm, and subsequently processed using a Savitzky–Golay smoothing filter (15 points). Baseline correction of the RNA spectra was conducted by subtracting spectra obtained from the buffers alone. Measured ellipticity in milidegree (mdeg) was converted to the molar ellipticity [*θ*] = deg × cm^2^ × dmol^−1^. Melting curves were measured at a constant wavelength in a temperature range of 10°C to 90°C with a sampling rate of 1°C/min. Melting curve data points were normalized and used for melting point calculations using the Equation (4) in OriginPro 2023: *A*1 is the lowest and *A*2 the highest normalized ellipticity, LOG_x1_ the first and LOG_x2_ the second melting point, *h*1 the slope 1, *h*2 the slope 2 and *p* the proportion (44).


(4) \begin{eqnarray*}y = A1 + \left( {A2 - A1} \right)\left[ {\frac{p}{{1 + {{{10}}^{\left( {{{{\rm LOG}}_{x1}} - x} \right)h1}}}} + \frac{{1 - p}}{{1 + {{{10}}^{\left( {{{{\rm LOG}}_{x2}} - x} \right)h2}}}}} \right]\end{eqnarray*}

### PD-MaP probing and data analysis

The 5′ UTR of the SARS-CoV-2 genome (stem–loops 1–5) with flanking structured cassette and barcode helices were prepared as described previously (18). Briefly, upon *in vitro* transcription, samples were purified via Mag-Bind beads (Omega), exchanged into H_2_O using 30-kDa molecular weight cut-off Amicon filtration units (Millipore), and diluted to 1 μM stocks for storage at 4°C. RNA samples were refolded by heating to 95°C for 2 min, followed by snap-cooling on ice for 5 min. 7.5 μl of the RNA sample was added to 10 μl of 2.5x buffering solution (750 mM sodium acetate at pH 5.0 or 750 mM bicine at pH 8.3, 150 mM KCl, 150 mM EDTA) and 5 μl of H_2_O, then incubated at 35°C for 20 min. DMS probing and controls were carried out by mixing 18 μl of the RNA sample with 2 μl of 1.7 M DMS in ethanol and neat ethanol, respectively, and incubating at 37°C for 6 min. Reactions were quenched with the addition of 20 μl of neat BME. Three replications were carried out at their respective pH conditions with one control (unmodified) sample. RNA samples were purified by buffer exchanging into H_2_O with 30-kDa MWCO Amicon filtration units (Millipore).

The mutational profiling (MaP) was carried out as described previously (45). Briefly, a mixture of 7 μl purified sample from the reaction, 1 μl of 2 μM RT primer, and 2 μl of 10 mM dNTPs was incubated at 65°C for 5 min and snap cooled on ice for 2 min. 9 μl of 2.22× MaP buffer (111 mM Tris at pH 8.0, 444 mM KCl, 45% glycerol, 11.1 mM DTT, 2.22 mM MnCl_2_) were added to the 10 μl mixture and incubated at room temperature for 2 min before adding 1 μl of MarathonRT reverse transcriptase (Kerafast) for reverse transcription. The resulting cDNAs were purified using 30-kDa MWCO Amicon filtration units (Millipore). A two-step protocol was employed to prepare cDNA sequencing libraries as described previously (45). The final cDNA libraries were purified with Mag-Bind beads (Omega), pooled, and sequenced on Illumina MiSeq instrument with a 2 × 150 cycle paired-end read kit.

Sequencing reads were merged using *BBmerge* (46). Replicates and pH-specific conditions were analyzed independently using *ShapeMapper2 (v2.2)* (47,48) to align reads to the reference sequence of the construct, detect mutations, and generate per nucleotide mutation rates above background for each pH condition. The pH differential mutational profiling (PD-MaP) analysis was performed to detect protonation signatures in the RNA as described previously (18). Secondary structure calculation was accomplished using *RNAstructure* with normalized DMS reactivity profile at pH 8 (49). Secondary structures were visualized using the online application of *RNAcanvas* (50).

## Results

### Solution NMR ensemble structure of SL1

**Figure 2.**
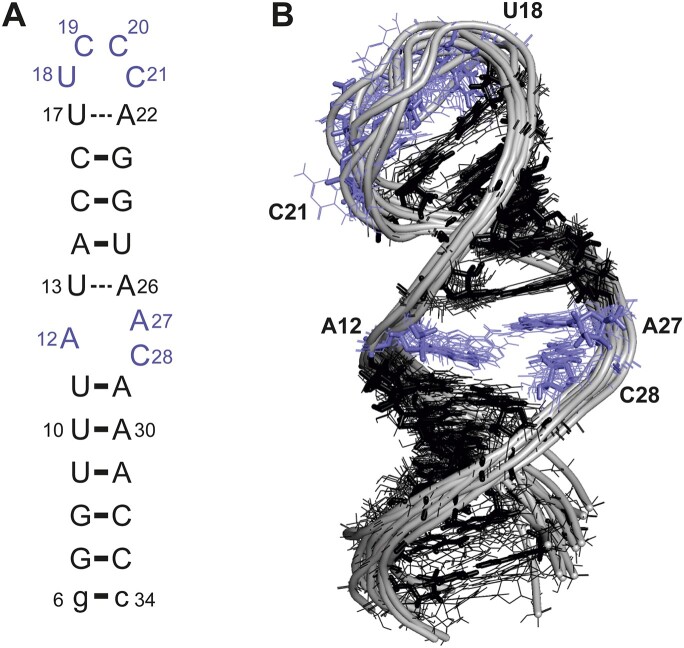
Structural ensemble of SL1. (**A**) Secondary structure representation of the SL1 construct investigated here. The additional G–C base pair added at the terminus for stability and transcription efficiency, which is not part of the original SL1 sequence is shown in lower case letters. Residues of the apical loop and the internal bulge are highlighted in violet. (**B**) NMR-derived structural ensemble of SL1 at pH 6.2. Individual structures are shown in thin stick representation with the backbone in cartoon representation; the lowest-energy structure is shown as thick sticks with the backbone in cartoon representation. Color scheme as in (A).

The sequence of SL1 and its secondary structure are shown in Figure 2A. In Wacker and Weigand *et al.*, we delineated structured and flexible parts of stem–loop SL1, showing that the apical loop region is highly dynamic, and closed by a labile Watson–Crick base pair (U17–A22). Furthermore, the U13–A26 base pair adjacent to the internal loop was also shown to be labile, but significantly populated. Both U–A base pairs exhibited ^*2*h^*J*_NN_ couplings >4 Hz indicative for partial hydrogen bond formation (15,27).

Here, we used our near-to-complete resonance assignment (BMRB entry 50349) to derive NOE distance restraints and semi-quantitative chemical-shift-based geometric restraints to calculate a structural ensemble for SL1. The final NMR-derived structural ensemble of SL1 comprises 10 conformational states (Figure 2B). The details of the NMR-input data and the structural statistics are summarized in Table 1. The ensemble has an overall r.m.s.d. of 1.8 Å.

**Table 1.** NMR and structural statistics for structure determination of SL1

NMR experimental restraints	
Total number of experimental restraints	747
Average no. of restraints per nucleotide	25.8
Average no. of NOE-derived restraints per nucleotide	12.8
NOE-derived restraints	372
Intra-residue	191
Inter-residue	181
Sequential |*i*-*j*| = 1	153
Non-sequential |*i*-*j*| > 1	28
Dihedral restraints	305
Hydrogen-bonding restraints	48
Planarity restraints	11
Residual dipolar couplings (RDCs)	11 (18)^a^
**Structure analysis of 10 lowest-energy structure bundle**	
av. *R²* RDC back-calculated versus RDC observed^a^	0.947
av. *χ²* to SAXS data^a^	3.37
all atom r.m.s.d. from mean structure, Å	1.79
**Structure analysis best model**	
*R²* RDC back-calculated versus RDC observed^a^	0.950
*χ²* to SAXS data	2.75

^a^18 residual dipolar couplings used for fit, 11 RDCs as restraints for ARIA calculation.

**Figure 3.**
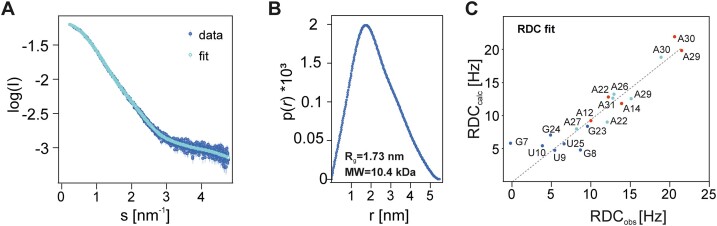
SAXS and RDC analysis of the SL1 structural ensemble. (**A**) Raw SAXS data and fit result of distance distribution. (**B**) Distance distribution function of curve shown in (A). (**C**) Calculated RDCs versus observed RDCs; blue: NH RDCs (not used in ARIA calculation); turquoise: C2H2 RDCs; red: C8H8 RDCs. The RDC fit is shown for the lowest-energy structure, *R*^2^ = 0.95. The most prominent outlier, G7, notably exhibits an elevated {^1^H}–^13^C heteronuclear NOE above 1.2.

We measured SAXS data to complement the information regarding general molecular shape and size of SL1 (Figure 3A, B). The trend observed for ensemble-fitting to the RDC data is reproduced by the SAXS data: CRYSOL fitting of SAXS data yields *χ²* = 3.37 using experimental SAXS data for values from 0.5 < *s* < 5.5 nm^−1^, omitting the large distances regime and potential artifacts arising from electrostatic intermolecular interactions or residual high-molecular-weight impurities. The individual lowest-energy NMR structure yields a *χ²* of 2.75 to this range of SAXS data. Analysis of *R_g_* and *D*_max_ (Supplementary Table S1) calculated from SAXS data using the GNOM software shows a tendency towards slightly smaller values than calculated for the NMR ensemble (Supplementary Table S1).

In summary, RDC and SAXS data are in excellent agreement with the solution structure (Figures 2 and 3).

### Structural characteristics of SL1

The well-defined A-form helical parts of SL1 comprise G6–C34 to U11–A29 and U13–A26 to U1–A22. These helical parts are connected by an asymmetric 1:2 internal loop formed by A12 on the 5′-part and A27 and C28 on the 3′-part and and capped by a UCCC-tetraloop. Superposition of A-form helical parts only of the NMR ensemble yields a r.m.s.d value of ∼0.75 Å for both stems. The internal loop-residues are consistently found in a stacked arrangement and overall A-helix-like conformation leading to a slightly kinked inter-helical angle between the upper and the lower stem of ∼150°, consistent with RDC and SAXS data (Figure 3). We wish to note that G7 exhibits an elevated hetNOE and the lower experimental RDC than its calculated RDC might reflect internal dynamics typical for helix termini (‘end fraying’) (Figure 3C, Supplementary Table S2) (51,52).

### The 4-pyrimidine-loop is disordered

**Figure 4.**
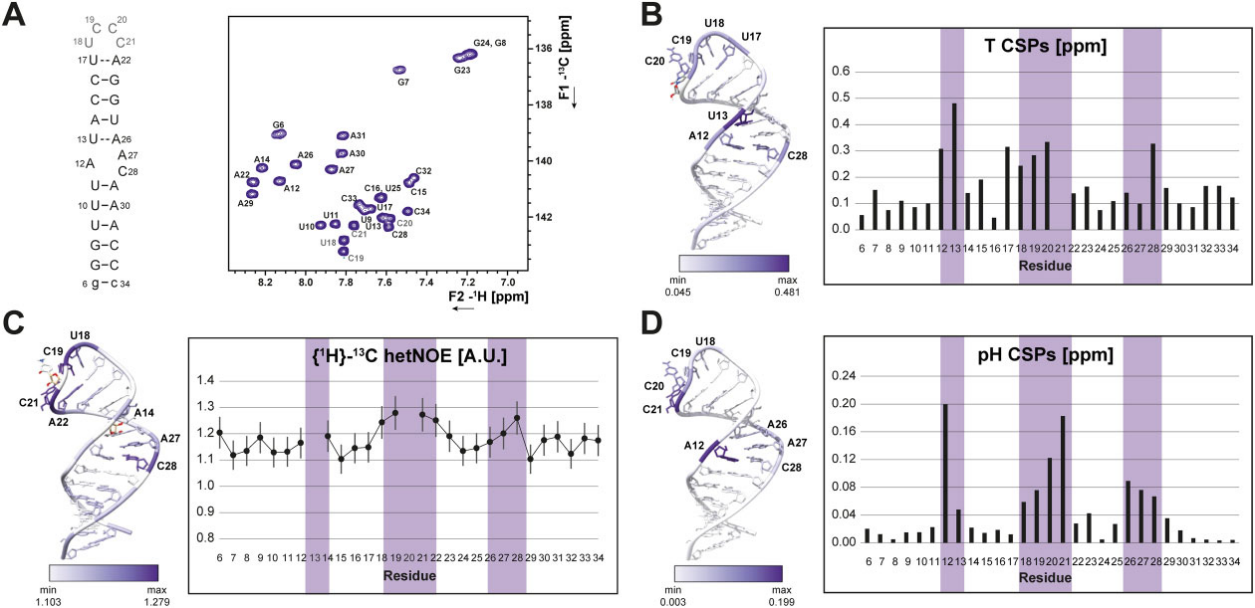
Changes in conformational dynamics of SL1 due to temperature and pH and analysis of local conformational flexibility. (**A**) ^1^H–^13^C-HSQC (H6/8-C6/8) at pH 6.2 and 298 K. (**B**) Temperature-induced CSPs, (**C**) {^1^H}–^13^C heteronuclear NOE values, (**D**) pH-induced CSPs plotted on the lowest-energy structure of SL1. Color bars corresponding to the range of respective values are given next to each structure. Values are shown for aromatic CH resonances between *T* = 278–308 K at pH 6.2 (B), *T* = 298 K and pH 6.2 (**C**), and pH = 5.2–6.2 at *T* = 283 K (**D**). Combined ^1^H,^13^C CSPs were used for the analysis shown in (B) and (C). Resonances could not be analyzed for residue 21 (B, T CSPs) and residues U13 and C20 (C, hetNOEs); these residues are colored according to their atom type.

The least defined part of the SL1 structural ensemble comprises the four nucleotides-spanning apical loop consisting of U18, C19, C20 and C21. 4-pyrimidine (4Y) tetraloops are not part of one of the stable classes of tetraloops (53), and a pdb search for 4Y-tetraloops yields a heterogeneous set of structures (Supplementary Table S3). The four loop nucleotides of SL1 show differences in residual structure and dynamics. The U18 and C19 C5H5 and C6H6 (Figure 4A) resonances are sharp and found at the downfield edge of typical chemical shift values in the ^1^H and the ^13^C dimension, indicating a lack of structure (54,55). Compared to U18 and C19, the chemical shifts of the nucleobase resonances for C20 and, to a lesser extent C21, are found in the more upfield spectral region, for which substantial contributions of stacking interactions define the proton chemical shift (27,56). The C6H6 chemical shifts of C20 and C21 are at the same time highly temperature-dependent (Figure 4B) and reveal a substantial increase in linewidths at temperatures below 288 K, prohibiting analysis of the complete CSP trajectory for C21. Accordingly, C20 and C21 undergo conformational exchange approaching the intermediate exchange regime on the NMR timescale at this temperature, while U18 and C19 exhibit sub-ns dynamics (27). The conformational flexibility in the apical loop is also reflected by {^1^H}–^13^C hetNOE values (Figure 4C), which allow more quantitative insight into the time scales of local motions. Despite their apparent different degrees of residual structure, three out of four loop residues exhibit elevated hetNOE values (C20 could not be analyzed due to overlap with U13) and thus experience internal motions faster than the molecular rotational correlation time (*τ*_C_), which is estimated to be ∼14 ns for SL1 at 298 K (57). In addition, the three loop cytidines show a marked pH response, reflecting the weak basic character of free cytidine imino groups (63) (Figure 4D).

The calculated structural ensemble generally captures these characteristics readily observable by NMR: In the apical loop region, the highest structural heterogeneity (r.m.s.d. ∼4 Å) is observed. However, since information about structural flexibility and chemical shift signatures are not easily incorporated into RNA structure calculations, and due to sparse NOE restraints for the apical loop residues, the four residues constituting the apical loop are predominantly defined by the force field employed in the structure calculation. Interestingly, the ensemble presents U18 as the least disordered residue of the loop (Figures 2 and 4C).

### The conformation of the internal loop is highly pH-sensitive

**Figure 5.**
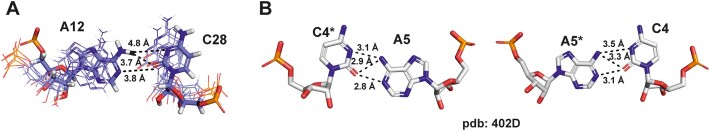
Close-up views of (**A**) the structural bundle of SL1 showing the internal loop region with focus on the proposed formation of the A^+^•C wobble base pair and (**B**) configuration of the two ideal A^+^•C wobble base pairs formed reported in pdb 402D (60). Asterisks denote the complementary strand of the duplex.

**Figure 6.**
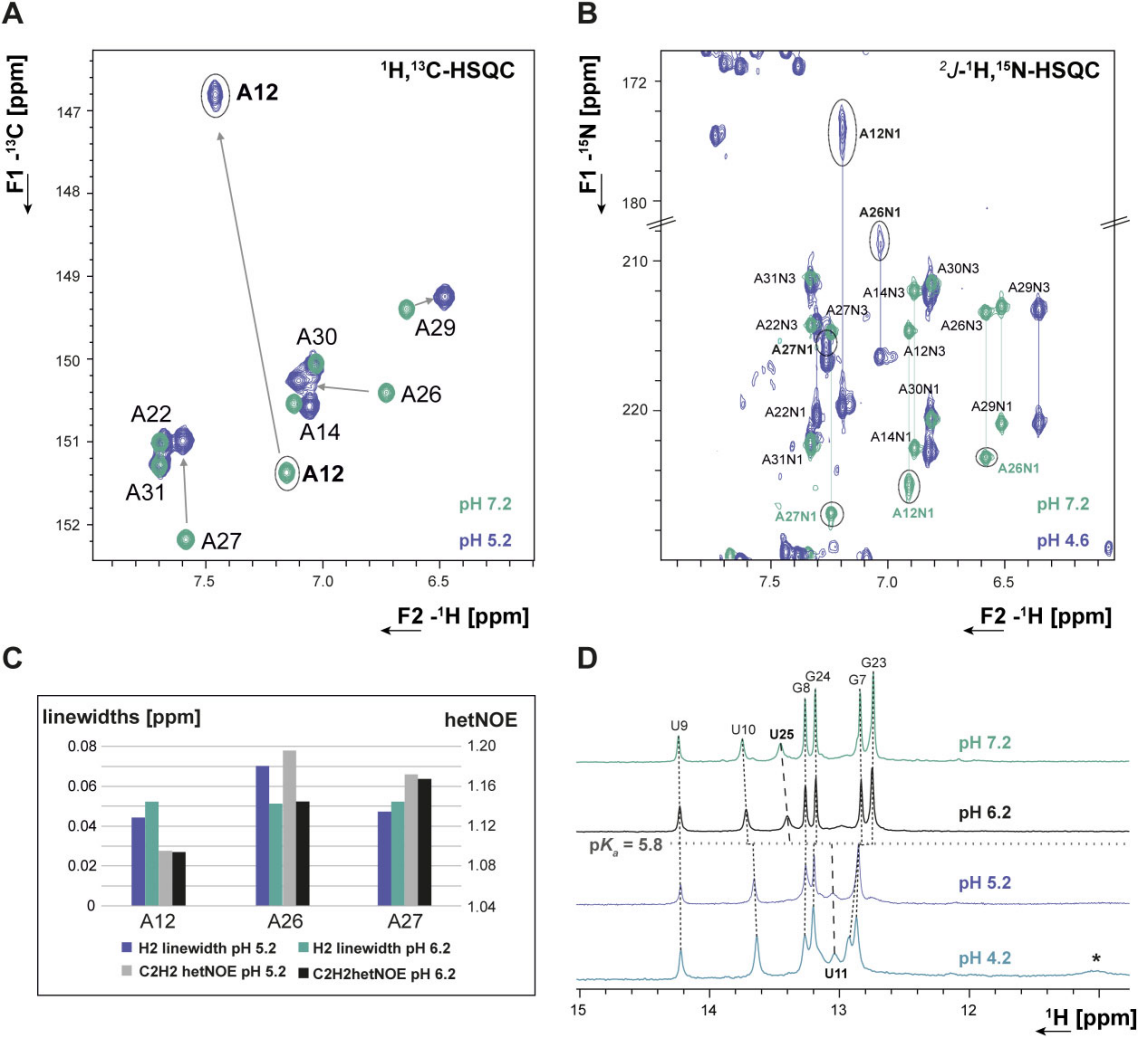
pH-sensitive conformational plasticity of the internal loop. (**A**) ^1^H,^13^C-HSQC spectra overlay at pH 7.2 (turquoise) and pH 5.2 (purple), the C2H2 resonance shifts for A12, A26 and A27 are indicated by arrows. (**B**) ^2^J-^1^H,^15^N-HSQC spectra overlay at pH 7.2 (turquoise) and pH 4.6 (purple) showing the pronounced upfield shift of the A12N1 resonance and significant upfield shifts for A26N1 and A27N1 resonances at lower pH, respectively. (**C**) Comparison of resonance linewidths and {^1^H}–^13^C hetNOE values of A12, A26 and A27 at pH 6.2 (turquoise/black) versus pH 5.2 (purple/grey). (**D**) 1D-^1^H NMR spectra showing the imino proton region of SL1 at different pH values. Opposite pH dependence of base pair stabilities reflected by imino proton intensities: Above the pK_a_ of A12, U13–A26 is most stable and a U25 imino resonance is observable. Below pH 5.8, U11 becomes observable due to stabilization of A12^+^•C28. (A, B) NMR spectra were acquired at 600 MHz and 298 K. (D) NMR spectra were acquired at 600 MHz and 283 K.

The residues A12, A27 and C28 forming the internal loop, are stacked between the lower and upper helical parts of SL1 in the structure ensemble at pH 6.2. This stacked arrangement of all three bulge residues is in line with upfield proton chemical shifts of their aromatic CH resonances and the presence of A-form typical sequential NOEs (58). Previous molecular dynamics simulations using state-of-the-art force fields have yielded a similar picture, where the nucleobase arrangements in the internal loop all preserve the coaxial stacking of the two helices (59). A striking feature of the NMR spectra for residues within this internal loop is that exclusively the N1 resonance of A12 remains undetected at any of our experimental conditions (27). Taking into account that the lowest-energy NMR structure shows A12 and C28 pre-arranged to form a *cis*WW wobble base pair (Figure 5A, Supplementary Table S4), we speculated that A12 might in fact be transiently protonated similar to the prototype A^+^•C base pair found in the RNA octamer characterized by Jang *et al.* (Figure 5B, Supplementary Table S4) (60). We therefore measured an ^1^H,^13^C-HSQC of SL1 at pH 5.2 in order to stabilize the putative protonated conformation. Indeed, at pH 5.2 and 298 K, the C2 resonance of A12 shows a significant upfield shift to <147 ppm compared to the listed mean value of 153.2 ppm (61), a reliable indicator for N1 protonation (Figure 4D and 6A) (22,62). We thus performed an NMR-detected pH titration. Fitting the pH-induced ^13^C CSPs of C2 atom of A12 as function of pH yielded a p*K*_a_ for A12N1 of 5.8 ± 0.2 (Supplementary Figure S1), corresponding to ∼28% population of protonated A12 species in the NMR structure. We further observed that only at pH values below 5.2, the A12N1 resonance becomes detectable at *δ*^15^N ∼176 ppm in *^2^J*-^15^N-HSQC spectra (Figure 6B, Supplementary Figure S2). Its chemical shift is still significantly downfield of the imino group resonances for stably protonated adenosines involved in hydrogen bonding interactions (22) and no significant further shift is detected below pH ∼4.7 (Supplementary Figure S2), which was also the stability limit we observed for our system. In line, no imino proton is observable for A12 even at *low* pH and low temperature. C4 and C2 chemical shift signatures of C28 at pH 5.2 show an intermediate state between stem and loop cytidines (Supplementary Figure S3). Thus, the A12^+^•C28 interaction remains transient even at *low* pH.

Interestingly, supercooled NMR spectra acquired at pH 4.8 in capillaries, initially recorded to find the A12^+^ imino proton, led to the detection of a novel imino resonance within the non-canonical range (Supplementary Figure S4). A ^1^H,^15^N-HSQC spectrum of SL1 at pH 4.2 and 278 K confirm this additional imino signal at 10.97/158.28 *δ*^1^H/*δ*^15^N ppm (Supplementary Figure S5A) to belong to a uridine involved in non-canonical interactions. The only observable NOE of this signal is to water (Supplementary Figure S5B), indicating that the imino proton experiences significant solvent exchange at *low* pH and low temperature. This new imino proton signal either belongs to U18, the first loop nucleotide, which might become solvent-protected upon pH-induced re-arrangement of the flexible loop residues. Alternatively, it belongs to U13, which at pH values <5.2 becomes released from the weak Watson–Crick base-pair with A26 present at higher pH (27). At a pH value of 5.2, the A26N1–U13N3 ^2h^*J*_NN_ coupling (27) is no longer observable, and the A26 nucleobase resonances exhibit significant downfield shifts along with increased linewidths (Figure 6C). These chemical shift signatures indicate that the formation of the A12^+^•C28 base pair is accompanied by the destabilization of the A26–U13 base pair. In this scenario, the U13 imino proton might be sufficiently solvent protected by stacking interactions or transient Hoogsteen-interactions with A27 at low temperature to exhibit a non-canonical imino chemical shift. The N1 resonances of both A27 and A26 are shifted upfield even with respect to their N3 resonances at *low* pH (Figure 6B), indicating that these imino nitrogens are susceptible to transient protonation. Especially A27 shows also a significant upfield shift of C2 (Figure 6C).

Next to the pronounced pH response detected for A12, A27 and A26 (Figure 6) at pH 5.2, the aromatic resonances of the loop residues C20 and C21 shift downfield (Supplementary Figure S3), reporting substantial changes in loop structure at *low* pH. The largest CSP is observed for C21, which appears to be released from partial stacking upon pH decrease. pH effects on most of the signals of the A-helical stems of SL1 are negligible (Figure 4D). We note here that the pH value of 4.2 is exactly the p*K*_a_ value for the N3 of all free cytidines (63). Thus, structural changes detected at lower pH values and those derived from the protonated A12 at this pH stem from different processes. Subsequently, the pH of 5.2 was chosen for the structural characterization of SL1, allowing stable protonation of A12 but avoiding complete protonation of all free cytidine residues.

Consistent with the *low* pH structural model (Figure 6A), although not stably formed, the labile A12^+^•C28 wobble configuration disfavors the A26-U13 interaction at pH < 6, while increasing pH and the loss of protonated A12 species leads to stabilization of the A26–U13 interaction.

At neutral pH, 6% of A12 residues remain protonated (Equation [2]). This is reflected in its downfield, canonical C2 and N1 chemical shifts (Figure 6A, B). Moreover, N1 resonances of the neighboring A26 and A27 residues are shifted downfield at pH 7.2 (Figure 6B). The imino proton signal of the internal loop-closing base pair U13–A26 is in fast solvent exchange throughout the tested pH range, but base-pair formation is unambiguously observed at pH 6.2 (27) and above. Accordingly, the A26 C2H2 resonance displays both reduced hetNOE and reduced linewidths at higher pH values (Figure 6C), indicating a loss of dynamics over a wide timescale. The opposite stability changes of A12^+^•C28 (most stable at *low* pH) versus U13–A26 (most stable at higher pH) is nicely reflected in the intensities of the imino proton resonances of the respective adjacent base pairs, where U25 is highest at pH 7.2 and U11 is highest at pH 5.2 (Figure 6D).

### Global stability of SL1 is increased at higher pH values

**Figure 7.**
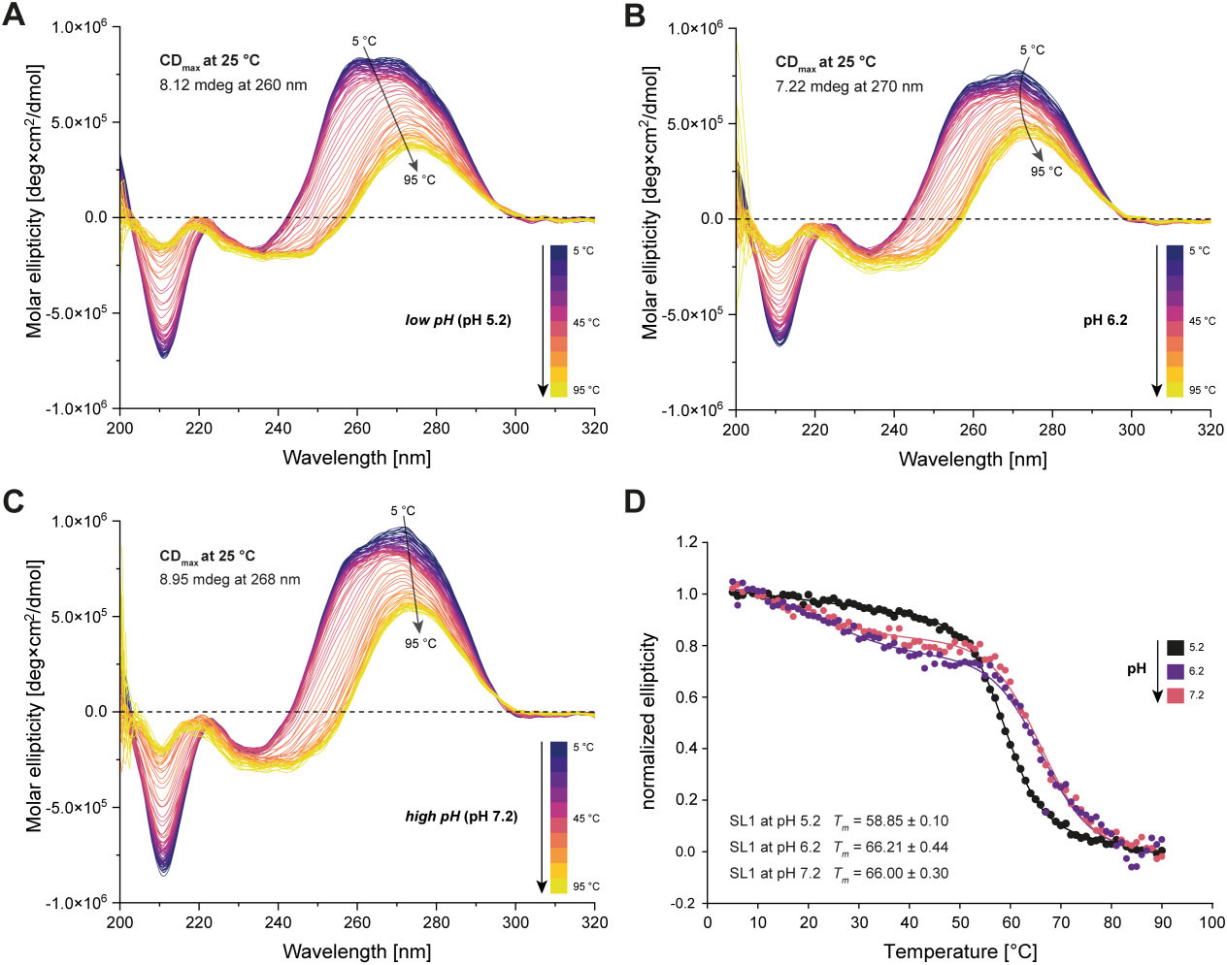
pH-dependent CD and melting curve analysis of SL1. (**A–C**) Overlay of CD spectra recorded in five accumulations at various temperatures (ranging from 5 to 95°C) with one-degree increments at pH 5.2, 6.2 and 7.2. Shifting of CD_max_ values are highlighted. (**D**) Melting curve analysis of SL1 at pH 5.2, 6.2 and 7.2. Data were extracted from recorded CD spectra recorded at 25°C from the wavelength belonging to the CD_max_ of the individual sample. Melting points were determined with a sigmoidal fit revealing increased global stability reflected in higher melting points at higher pH (*R*^2^_pH 5.2_ = 0.9994, *R*^2^_pH 6.2_ = 0.9905, *R*^2^_pH 7.2_ = 0.9958).

CD spectroscopic measurements conducted at various temperatures and pH values allowed accessing the impact of pH on the global stability of SL1 (Figure 7). Analysis of melting curves consistently revealed higher melting temperatures in line with enhanced thermal stabilization of secondary structure with increasing pH, as evidenced by the shift towards higher wavelengths of the maximal CD signal (Figure 7B, C). Notably, protonation and subsequent transient A12^+^•C28 interaction at *low* pH levels decreases the global stability, resulting in a reduced melting point (Figure 7D). This would be expected, given the still transient interaction reflected in the larger distances of H-bond donor and acceptor groups compared with a stably formed A^+^•C wobble base pair (see Figure 5).

A more detailed examination of individual CD curves across different pH values suggests the presence of multiple states at lower temperatures and pHs of 6.2 and 7.2. This is particularly evident in the change in line shape between 260 and 280 nm as temperatures increase, primarily affecting the detection of the CD maximum. We classify maxima located in the previously mentioned region as different secondary structures due to their behaviour at higher temperatures. At higher temperatures, CD curves show a clear ‘one-state’ maximum as most of the base pair interactions and therefore secondary structures are disrupted at these temperatures. For example, the CD profile observed at pH 6.2 is indicative of two distinct states at lower temperatures compared to the curves at pH 5.2 and 7.2. Notably, *high* and *low* pH conditions exhibit a greater tendency towards a specific state, highlighting pH-dependence in the structural dynamics of SL1.

In general, the collected CD data confirm the presence of A-form helical structures in SL1. This is evidenced by the positive molar ellipticity observed at 260 nm and the negative ellipticity recorded around 210 nm (64). The CD data support our NMR results: the reduced CD-derived melting point of the *low* pH sample indicates that protonation of the internal loop coincides with the destabilization of the A26–U13 interaction. Both the upper and lower stems of SL1 experience compromised stability due to transient A^+^•C formation, while overall stabilization is regained at higher pH levels. Additionally, first and second order derivations showed a completely different pattern for SL1 at pH 6.2 when plotted against temperature (Supplementary Figure S6). This also agrees with our NMR results, as we assume a dynamic equilibrium between two distinct states of the internal loop at this pH environment.

### The SARS-CoV-2 5′-UTR contains multiple pH-responsive sites

**Figure 8.**
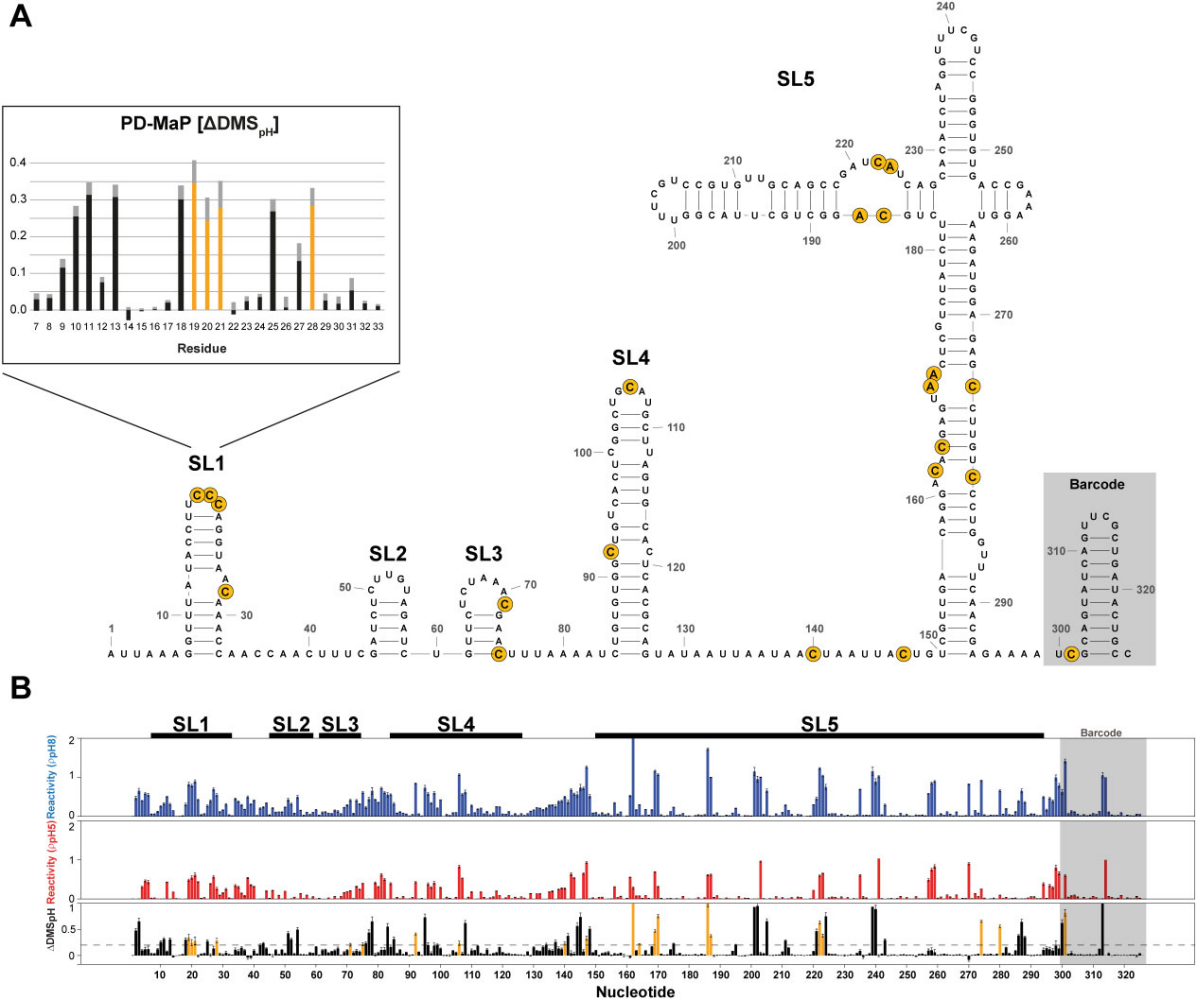
PD-MaP of SL 1-5 of SARS-CoV-2 5′-UTR. (**A**) Secondary structure of SL 1-5 of SARS CoV-2 5′-UTR with protonation signatures highlighted in yellow with focus on SL1’s PD-MaP profile. (**B**) Average PD-MaP profiles over three replicates. Protonation signatures are highlighted in yellow on the ΔDMS_pH_ plot.

Our high-resolution NMR characterizations have identified two independent, pH-sensitive motifs, here in SL1 and previously published for SL4 of the SARS-CoV-2 5′-UTR (17). To evaluate the prevalence of pH-sensitive conformational transitions in other structural regions of the SARSCoV-2 5′-UTR, we applied PD-MaP with dimethyl sulfate (DMS) on a construct encapsulating stem–loop 1–5 (Figure 8, Supplementary Tables S5 and S6) (18). Differential reactivities, ΔDMS_pH_ = ρ_pH8_ – ρ_pH5_, were generated from internally normalized DMS reactivities at *high* and *low* pH to highlight adenines and cytosines that exhibit pH-dependent solvent accessibilities at their Watson–Crick interfaces as a result of either direct protonation at their N1/N3 sites or structural changes due to proximal protonation events. Probing experiments were performed in triplicates with highly reproducible, internally normalized reactivities (Supplementary Figures S7, S8). The resulting PD-MaP profiles for the SARS-CoV-2 5′-UTR are shown in Figure 8, with protonation signatures highlighted. The presented secondary structure is determined using the normalized per nucleotide reactivities from the pH 8 dataset, which agrees well with the in-cell probed structure determined recently (Supplementary Figure S9) (17).

Strikingly, our PD-MaP results for the SARS-CoV-2 5′-UTR comprising stem–loops 1–5 show multiple sites of pH-affected reactivity, indicating local structural sensitivity of the 5′-UTR stem–loops towards pH. SL1 shows four PD-MaP-sensitive nucleotides, the three cytidine residues of the apical loop (C19, C20, C21) and C28 located in the asymmetric internal loop (Figure 8). These results confirm our previous findings regarding the transient A12 protonation: In its protonated state, A12 would serve as a hydrogen bond donor, thereby likely altering the accessibility of C28’s WC-site, as evidenced now by PD-MaP.

Across the 5′-UTR, the protonation signature hits are mostly prominent in the AC-rich internal loops of SL5, suggesting protonation-coupled conformational changes are present in multiple untranslated regions of the viral genome. We also noted that protonation signature hits were observed for several residues in the unstructured regions. While the secondary structure is determined at pH 8, the underlying structural properties of these unstructured regions at *low* pH are subject to future investigations. Overall, we have observed multiple protonation-coupled structural changes throughout the SARS CoV-2 5′-UTR, suggesting cellular acidity could modulate its conformational ensemble.

### The internal loop of SL1 can be targeted by small molecules

Previous fragment-based screening of SARS-CoV-2 derived structural RNA elements revealed five fragments from of a poised library containing 768 diverse fragments to bind to SL1 (14). Binding characteristics of these five small fragments were further investigated with follow-up NMR experiments, both ligand- and RNA-observed. Here, we highlight fragment C11, *N*-(4-methoxyphenyl)-2-(pyrrolidin-1-yl)acetamide, which showed the most promising results from the selected fragments. At *low* pH, C11 itself is present in a protonated state which excludes reliable experiments at pH 5.2 (Supplementary Figure S10A). Due to this protonation of ligand C11 and the pH sensitivity of A12 at pH 6.2, we decided to perform NMR- and 2AP-measurements with C11 only at pH 7.2.

**Figure 9.**
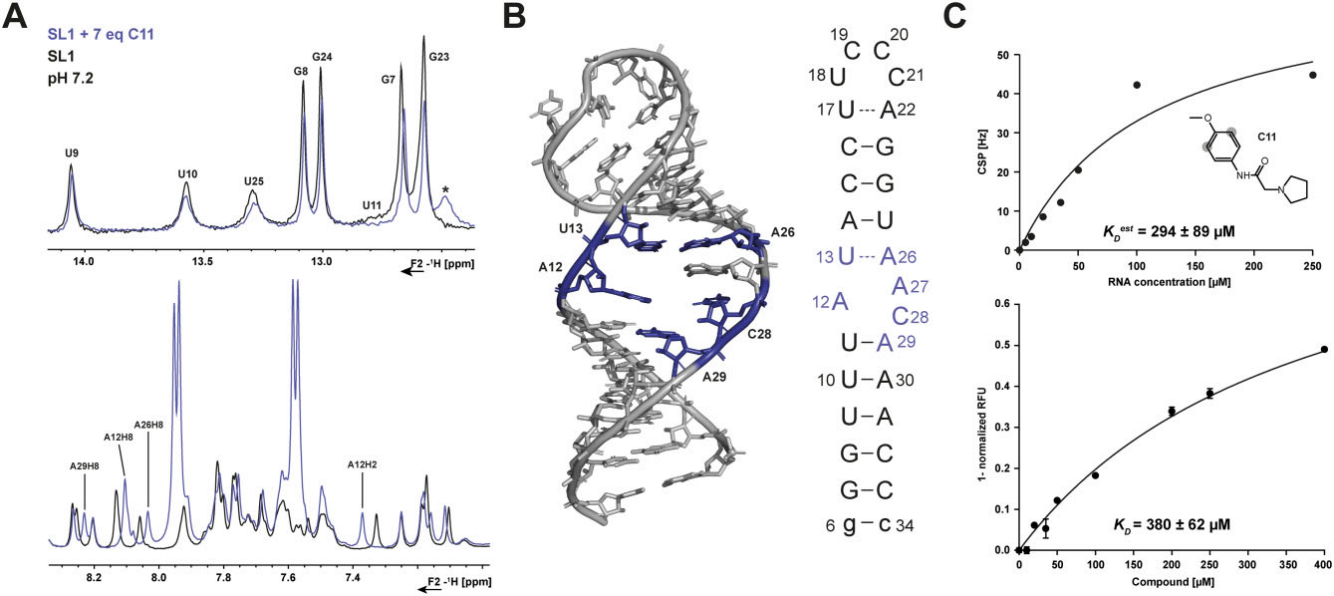
Characterization of the interaction between SL1 and C11 at pH 7.2. (**A**) RNA-observed chemical shifts induced by fragment addition. The addition of C11 to the target RNA allows the detection of an additional imino signal (top, *). Shifts within the aromatic region indicate binding of C11 to the internal loop of SL1 (bottom). The ligand was added in excess with an [RNA]:[ligand]-ratio of 1:7 (150 μM:1 mM). NMR spectra were acquired at 600 MHz and 298 K. (**B**) Mapped major CSPs (blue) induced by the addition of C11 to the tertiary structure identifying the internal loop as the binding site. Shifts taken from 1D- and additional 2D-NMR experiments shown in the SI. (**C**) Ligand-observed NMR titration fit (protons chosen for CSPs observation are indicated in grey) with the respective dissociation constant (top, *R*^2^ = 0.99975). RNA-observed fluorescence-based titration fit with the respective dissociation constant (bottom, *R*^2^ = 0.99926).

RNA-observed NMR measurements were applied to map the binding site of C11 (Figure 9A, B, Supplementary Figure S10B). Upon addition of C11, an additional imino signal was detected in the imino region of the RNA at around 12.5 ppm indicating a newly formed H-bond (Figure 9A, top). Intermolecular NOEs between SL1 and C11 were, however, not detected in NOESY experiments even after optimizations including different NOESY mixing time, temperatures, and different [ligand]:[RNA]-ratios (data not shown here). The signal overlap of the aromatic resonances of SL1 and the compound C11 introduces a bias in the analysis of binding-induced shifts of the RNA resonances, such as A27. However, low-affinity binding of C11 to the internal loop of SL1 is apparent even in 1D-^1^H spectra and can be assigned to some extend in the aromatic region of the spectrum (Figure 9A, bottom). Additionally, the 2D-^1^H,^1^H-TOCSY experiment reveals similar results: H5H6 chemical shifts of pyrimidines located in and neighboring to the internal loop restrict the binding site to the internal A12:A27C28 loop and the U11-A29 base pair of the lower stem as well as the one of the predicted U13-A26 base pair of the upper stem (Figure 9B, Supplementary Figure S10B). Additionally, we measured ligand-based NMR titrations in which the concentration of C11 was kept constant whilst SL1 concentration was increased (Figure 9C, top). Tracing the aromatic protons of C11 across eight different NMR spectra and determining the CSPs allowed us to estimate a *K*_D_ of 294 ± 89 μM at pH 7.2 (Supplementary Figure S10C). We note that these titration data estimate the dissociation constants since the NMR ligand-based titrations cannot report on the actual *K_D_*with high precision as its determination is biased by line broadening based on the exchange regime. The more RNA is added to the constant ligand concentration the more line broadening of the observed ligand peaks appears which influences appropriate peak picking. Besides, concentration ratios are limited due to the solubility characteristics of the used compounds. For further validation, we determined the *K*_D_ with an additional approach, namely an RNA-based fluorescence titration (Figure 9C, bottom). We applied 2AP labeling of SL1 at the internal loop at position 27, which allowed monitoring of alterations in the intrinsic fluorescence upon the addition of C11 at the assumed binding site. This labeling method is commonly used to observe RNA in a spectroscopic manner when e.g. investigating binding events or folding dynamics (67–69) (Supplementary Figure S10D). Using this approach, we determined a *K*_D_ of 380 ± 62 μM at pH 7.2. This value falls within the same range as the estimated NMR-based *K*_D_, taking into account the error values.

## Discussion

We report here the second solution NMR structure of a SARS-CoV-2-derived RNA element. The NOE-based structural ensemble is consistent with orthogonal experimental RDC and SAXS data.

The determination of a high-resolution structure allows important follow-up experiments. As an example, we use the structure to determine the major binding epitope for low molecular weight binders to SL1. This structural mapping of the RNA binding epitope provides an important guide for follow-up medicinal chemistry campaigns that are ongoing in our and other labs. Importantly, we show that SL1 features a pH sensitive restructuring of the internal loop and thus its major binding site, accompanied by transient non-canonical base pair formation. This pH-responsiveness needs to be taken into account, e.g. in virtual screening campaigns as well as docking and molecular dynamics (MD) experiments. Interestingly, also other RNA elements of the viral 5′-UTR, like SL4 and SL5, feature responses to changes in pH, raising the question of the relevance of pH at different stages of the viral life cycle. Indeed, viral replication and transcription is localized to double-membrane-vesicles (DMV) and also at later stages including packaging and viral egression, the respective environmental conditions become increasingly acidic (70–72). Beyond, structural data will support further investigations of the proposed SL1–Nsp1 interaction in which the pH-sensitivity of SL1 needs to be taken into account. Consideration must also be given to protonation characteristics during drug development. We revealed that differences in pH strongly affect binding of C11 to SL1, which adds another point of view regarding drug discovery when targeting SL1.

**Figure 10.**
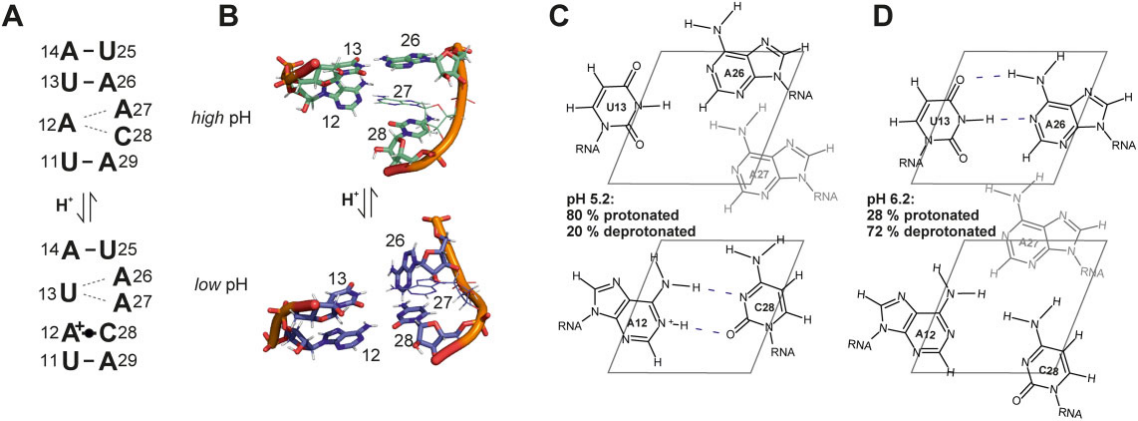
pH-induced re-arrangement of the internal loop upon A12 protonation. (**A, B**) A12 protonation leads to the stabilization of the A12–C28 interaction and to the destabilization of the U13–A26 interaction. (**C**) At pH 5.2 and below, the A12^+^•C28 wobble base pair is formed and A27 is sandwiched between U13 and A26 and A12^+^•C28. (**D**) At pH 6.2 and above, the U13–A26 base pair is formed and A27 is sandwiched between A12 and C28 and U13–A26. Under physiological conditions, the population of the protonated state decreases to 6%.

Comparison with accessible populations suggested by MD trajectories (59) supports a picture of internal loop dynamics, switching between different states. Our NMR-based experiments here show a clear pH-dependency of these different states and should expand our understanding of local structure changes induced by moderate environmental parameters, such as pH changes within physiologically relevant ranges. We further demonstrate that these subtle rearrangements of the internal loop allosterically influence the stability of the upper stem (Figure 10), similar to the protonation-induced maturation of miRNA-21 prior to Dicer cleavage (23) or the A^+^•C stabilization of miRNA-31 (73). The destabilization caused by the formation of the A12^+^•C28 base pair may appear counterintuitive at first glance. However, the transient nature of this base pair is evident even at *low* pH, which affects the stacking interactions of neighbouring base pairs, leading to global rearrangements and resulting in loss of global stability (74,75). Thus, our findings demonstrate that the transient interaction at *low* pH has the unexpected consequence of destabilizing the global structure of SL1, contrary to cases where stable protonation leads to a stable global RNA architecture (76). Moreover, a comparative analysis was conducted on the C2H2 and C8H8 hetNOE values of adenosine residues within SL1. The investigation revealed greater flexibility for the residues A12 and A29 under conditions of elevated pH, contrasting with enhanced flexibility observed for A26 under *low* pH conditions (Supplementary Figure S11). These local effects of pH on single nucleotide flexibilities are complemented by our CD data, showing that at *low* pH, globally destabilizing effects are dominant.

We used our structural model of the internal loop to compare it to published structures built by similar nucleotide sequences. The results of our webFR3D and RNA CoSSMos motif searches to scan the pdb for internal 1:2 loops composed of A:AC or, more generally, P:PY are shown in Supplementary Figure S12 (77,78). Interestingly, an alternative conformation, with an A:A mismatch and Y’28’ bulged-out, is adopted by the so-called apoB mRNA (54). This RNA element is a target site for a cellular cytidine-deaminase, one of the RNA editing enzymes also discussed to play a role in SARS-CoV-2 host adaption (79). In summary, the motif search revealed that a fully stacked conformation of these 1:2 internal loops was rarely reported so far (Supplementary Figure S12, Supplementary Table S3). The transient pre-organization of the SL1 internal loop promotes the formation of an A12^+^•C28 wobble base pair upon A12 protonation by structurally stabilizing the protonated form of A12, following a general principle suggested by Eaheart *et al.* via thermodynamic analysis (80). Consequently, the internal loop resembles motifs bearing a G•U at the positions corresponding to A12 and C28 of such 1:2 internal loops, as reported for the 25S rRNA of *Saccharomyces cerevisiae* S288C (PDB code: 5MC6; Supplementary Figure S12) (81).

Already in 2020, experimental data have been published on the secondary structure of the structured elements of the SARS-CoV-2 RNA genome by NMR spectroscopy (15) and structural probing (66,82–85), soon followed by 3D structure models derived by FARFAR2 (86), all-atom molecular dynamics (59) and coarse-grained modelling methods (87–89). While experimental secondary structures usually rely on computational prediction, those prediction tools are so far unable to include non-canonical base pairs except for G•U/U•G wobble base pairs, nor environmental effects such as ionic conditions or differences in pH (90). Given that the 16 experimental secondary structures of SARS-CoV-2 RNAs determined by NMR spectroscopy unambiguously identified 10 pyrimidine-pyrimidine mismatches in addition to 11 bulges, 13 internal loops and helix junctions, and 17 apical loops of varying sizes (14,15), we expect that further detailed structural studies will reveal more realistic 3D structure models, taking into account conformational dynamics and high-resolution structural information. With regard to virtual screening campaigns or *in silico* prediction of potential host interaction partners, especially these RNA regions of conformational plasticity are often crucial and need to be accurately described. For known RNA structures, microscopic p*K*_a_-values can be computed (91). Efforts are ongoing to include protonation events into RNA structure modelling by the use of polarizable force fields (92,93) or continuous constant pH molecular dynamics (94). Especially RNA structures that are transiently protonated instead of featuring stabilized hydrogen-bonded protonated residues such as the preQ1 aptamer (95) and the c-di-GMP (22) binding riboswitch aptamer domains or the artificial neomycin riboswitch (96) may often escape identification, and structural consequences of transient protonation may not be easy to take into account.

As an outlook, the determination of a high-resolution structure of SL1 at *low* pH employing emerging polarizable force fields will provide insight into structural principles for p*K*_a_ shifting, allowing prediction of protonation sites in RNA structures in near future and in understanding pH-induced structural rearrangements in biological context.

## Supplementary Material

gkae477_Supplemental_File

## Data Availability

Chemical shifts are deposited at BMRB under entry number 50349. Structural coordinates are deposited at wwPDB with the deposition ID: 9EOW. SAXS data were submitted to the SASBDB with the deposition ID: SASDSP7. PD-MaP sequencing data are deposited at SRA with the deposition ID: PRJNA1111894.

## Notes

**Dedication:** We dedicate this work to Prof. Hashim Al-Hashimi at the occasion of his 50th birthday.
