# Supplementary material for: The 5′-terminal stem–loop RNA element of SARS-CoV-2 features highly dynamic structural elements that are sensitive to differences in cellular pH
Source: Nucleic Acids Res. 2024 Jun 6;52(13):7971–86. doi: 10.1093/nar/gkae477 (PMC11260494; doi:10.1093/nar/gkae477)
Supplement: gkae477_Supplemental_File [file gkae477_supplemental_file.pdf]

Supplementary Information

The 5'-terminal stem-loop RNA element of SARS-CoV-2 features highly dynamic structural elements that are sensitive to differences in cellular pH

AUTHORS

Sabrina Toews[1,2,†], Anna Wacker[1,2,†], Edgar M Faison[3], Elke Duchardt-Ferner[2,4], Christian Richter[1,2], Daniel Mathieu[5], Sandro Bottaro[6], Qi Zhang[3], Harald Schwalbe[1,2,*]

[1]Institute of Organic Chemistry and Chemical Biology, Johann Wolfgang Goethe-University Frankfurt, Frankfurt/Main, Hesse, 60438, Germany

[2]Center for Biomolecular Magnetic Resonance (BMRZ), Johann Wolfgang Goethe-University Frankfurt, Frankfurt/Main, Hesse, 60438, Germany

[3]Department of Biochemistry and Biophysics, University of North Carolina at Chapel Hill, Chapel Hill, NC, 27599, USA

[4]Institute of Molecular Biosciences, Johann Wolfgang Goethe-University Frankfurt, Frankfurt/Main, Hesse, 60438, Germany

[5]Bruker BioSpin GmbH, Ettlingen, Baden-Württemberg, 76275, Germany

[6]Linderstrøm-Lang Centre for Protein Science, Department of Biology, University of Copenhagen, 2200, Denmark

[†]Joint First-Authors

*To whom correspondence should be addressed. Tel: +49 69 798 29737; Fax: +49 69 798 29515, Email: schwalbe@nmr.uni-frankfurt.de

The work is dedicated to Prof. Hashim Al-Hashimi at the occasion of his 50th birthday

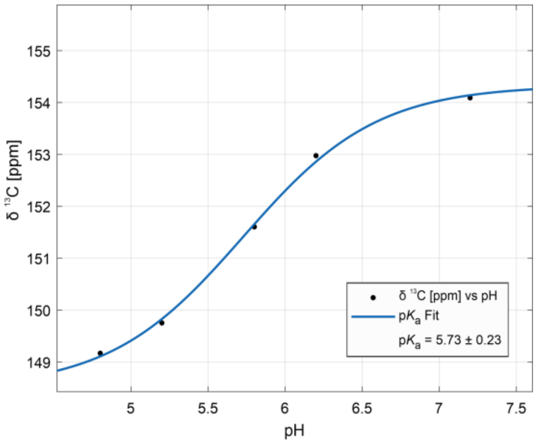

Figure S1, related to figure 4 *pK_a plot based on the pH-induced ^{13}C-CSPs of the A12 C2 resonance reveals a pK_a of ~5.8 for the A12N1. The carbon chemical shift was measured using an ^{1}H,^{13}C HSQC optimized for the aromatic resonances as described (1) at 600 MHz and 298 K.*

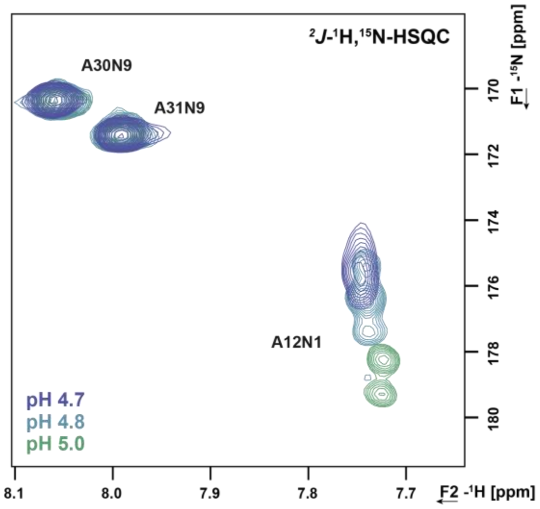

Figure S2 *pH-dependent A12N1 shifts: The A12 H2N1 resonance was monitored in ^{2}J-^{1}H,^{15}N-HSQC spectra at pH values from 5.0 (green) to 4.7 (purple). The A12N1 resonance shifts towards the ^{15}N spectral region of canonical H8,N9 chemical shift ranges for adenosine. The experiments were recorded at 800 MHz with 16 scans and a spectral width of 9.8 x 32 ppm and 1024 x 192 complex points in the direct and indirect dimension, respectively.*

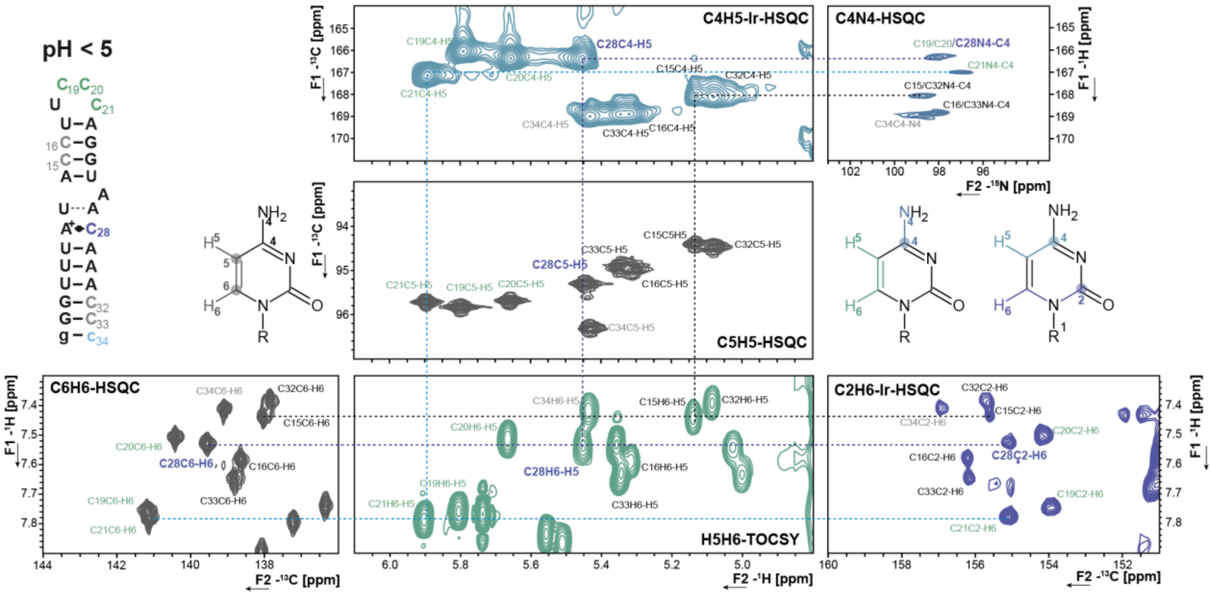

Figure S3 *Chemical shift signature of C28 at pH <5. NMR data were acquired at 600 MHz and 298 K. Correlations are represented by the respective color code highlighted within the cytosine structures: loop cytidines: green; stem cytidines: black; C28: purple; C34: light grey* (2–5).

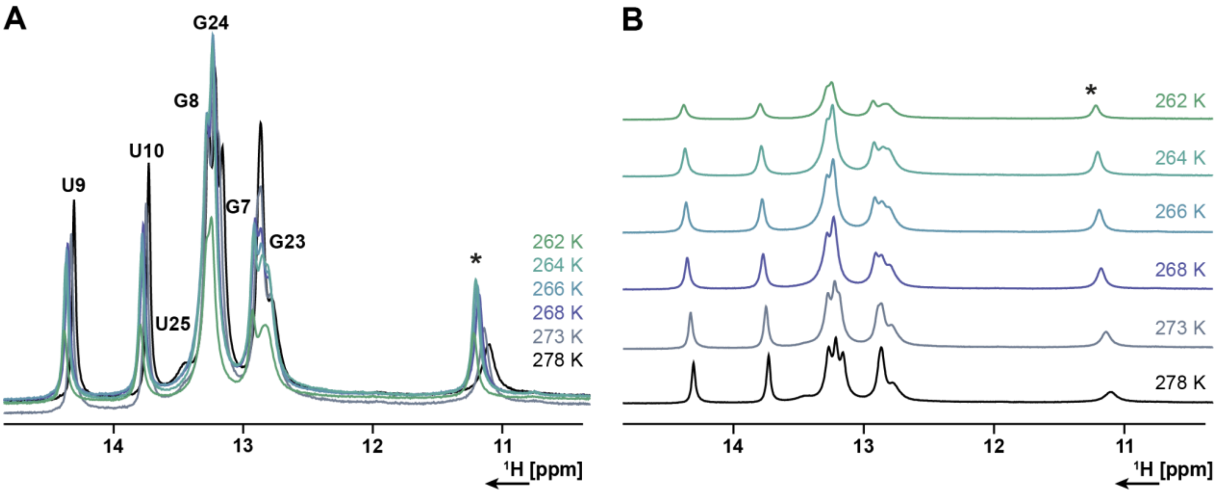

Figure S4 *Supercooled NMR experiment of an unlabeled SL1 RNA sample allows the detection of an additional imino signal at a pH of 4.8.* ***A*** *Overlay of the imino region of 1D ^{1}H spectra recorded at pH 4.8 and different temperatures. Chemical shift assignments are annotated. An additional imino signal is observed around 11.2 ppm, indicated by an asterisk.* ***B*** *Stacked view into the imino ^{1}H chemical shift region at different temperatures. Asterisk highlight the novel detected imino signal. A total concentration of 840 µM SL1 was measured in 1 mm capillaries at pH 4.8. NMR data were acquired at 700 MHz and in a temperature range between 262 and 278 K* (6, 7).

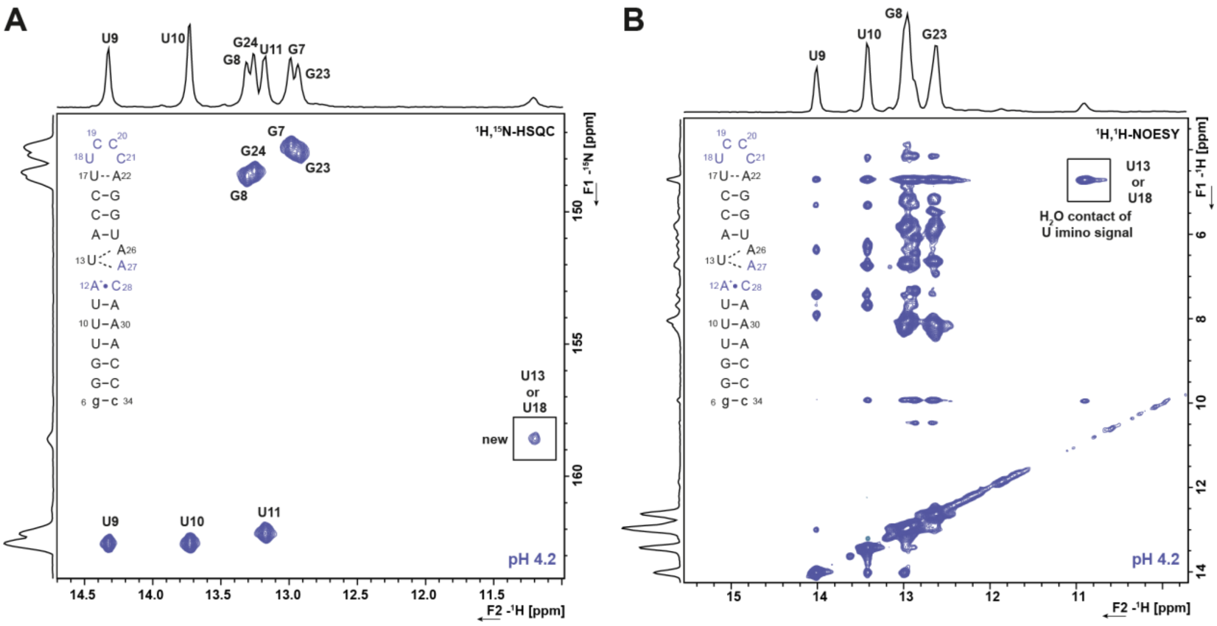

Figure S5 *Detection of the U imino signal appearing at lower pH.* ***A*** *2D-^{15}N,^{1}H-HSQC spectrum leads to the detection of a new non-canonical U imino signal at 10.97/158.28 δ^{1}H/δ^{15}N ppm at lower pH.* ***B*** *2D-^{1}H,^{1}H-NOESY (mixing time = 100 ms) shows that the novel imino signal only has exchange peak to water. This exchange is less pronounced than exchange peaks of G8 and G23 indicating reduced solvent exchange rates for this new peak under the applied conditions compared to unstructured residues. NMR spectra were acquired at 800 MHz, 278 K and a buffer pH of 4.2.*

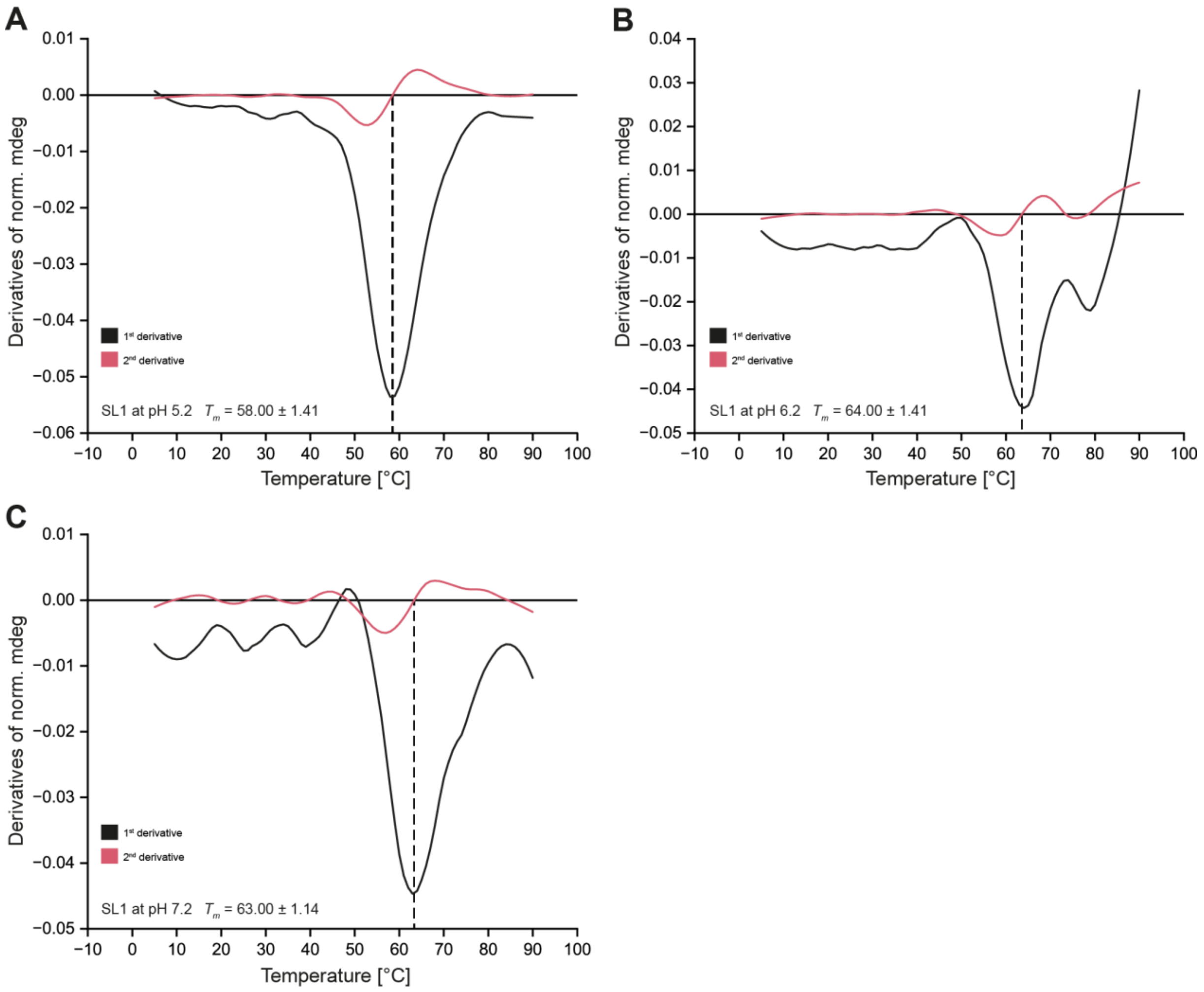

Figure S6, related to figure 7 *pH-dependent melting point determination of SL1 by using derivations of the CD melting curves. Data were smoothened by applying a Savitzky-Golay filter with 15 points before the first and second derivation. Melting points were taken at the minimum of the 1st derivation curve at that point where the curve if the 2nd derivation crosses zero. Standard deviation was calculated from ± two points around the melting point. Data were recorded at pH 5.2 (**A**), pH 6.2 (**B**) and pH 7.2 (**C**).*

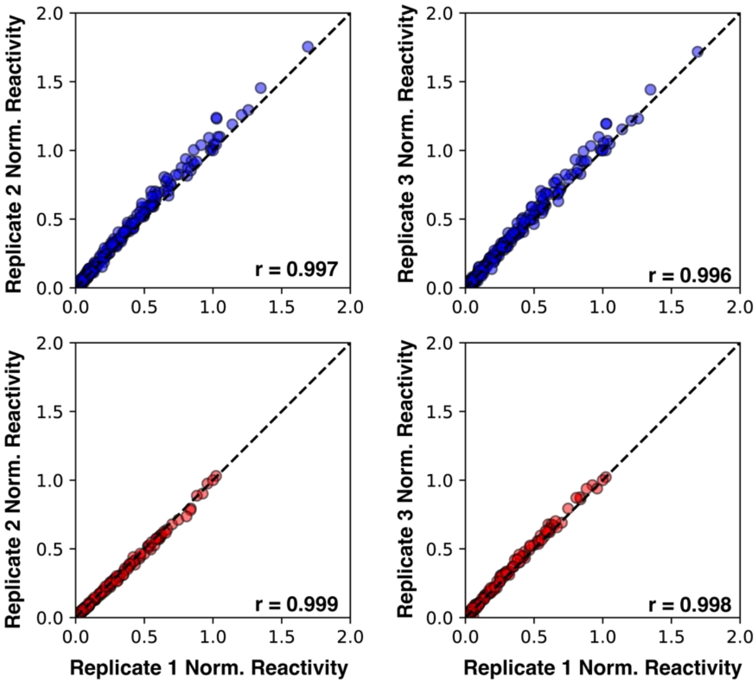

Figure S7, related to figure 8 *Correlation plots comparing replicates of DMS probing for pH 8.0 (top, blue) and 5.0 (bottom, red) sets.*

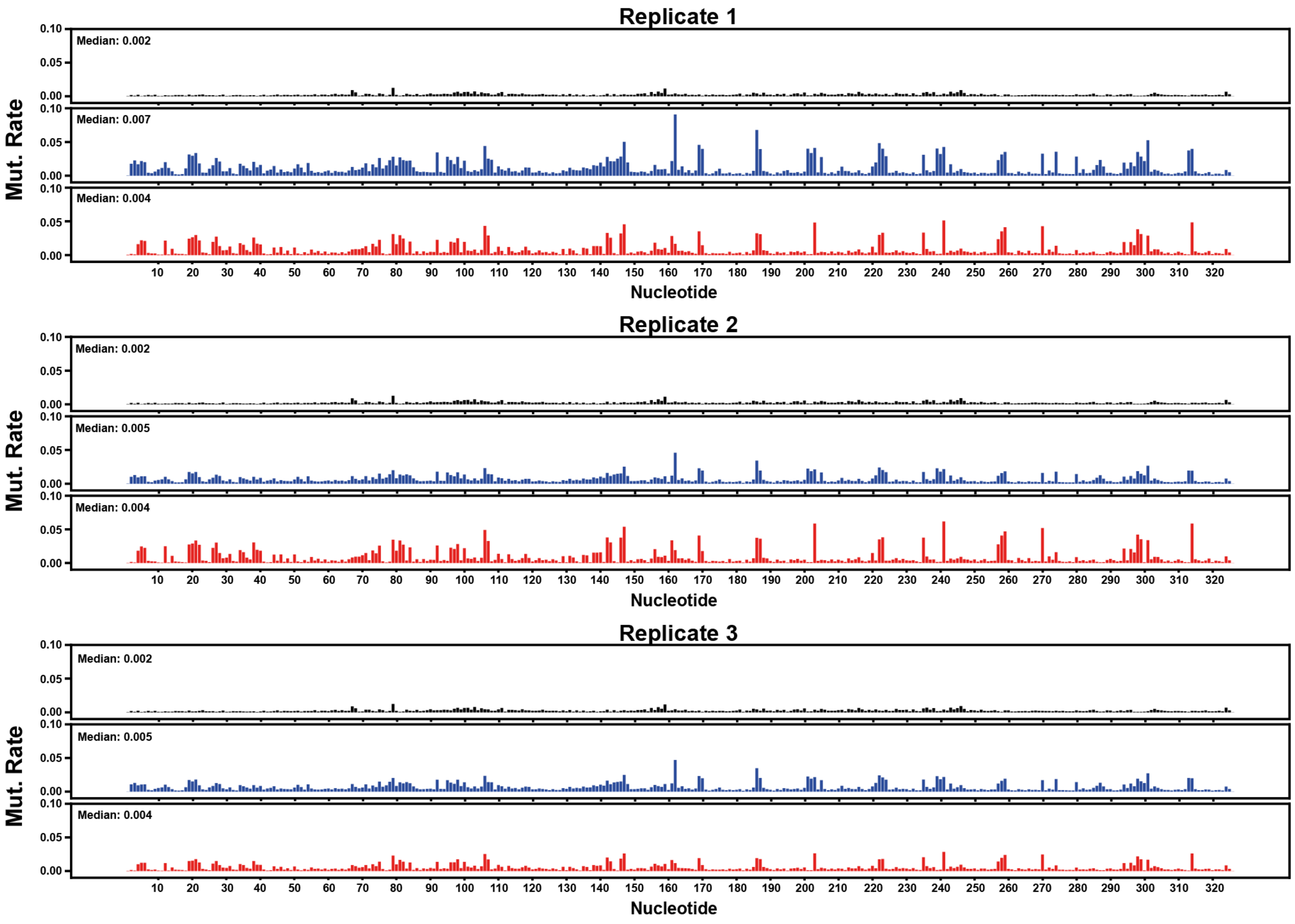

Figure S8, related to figure 8 *Raw mutation rates for the control (-DMS), pH 8 (+DMS), and pH 5 (+DMS) data for each replicate with median mutation rates for each set listed in the upper left-hand corner. Control samples generally exhibit lower mutation rates compared to +DMS samples at either pH, with pH 8 exhibiting the greatest median reactivity in each replicate set.*

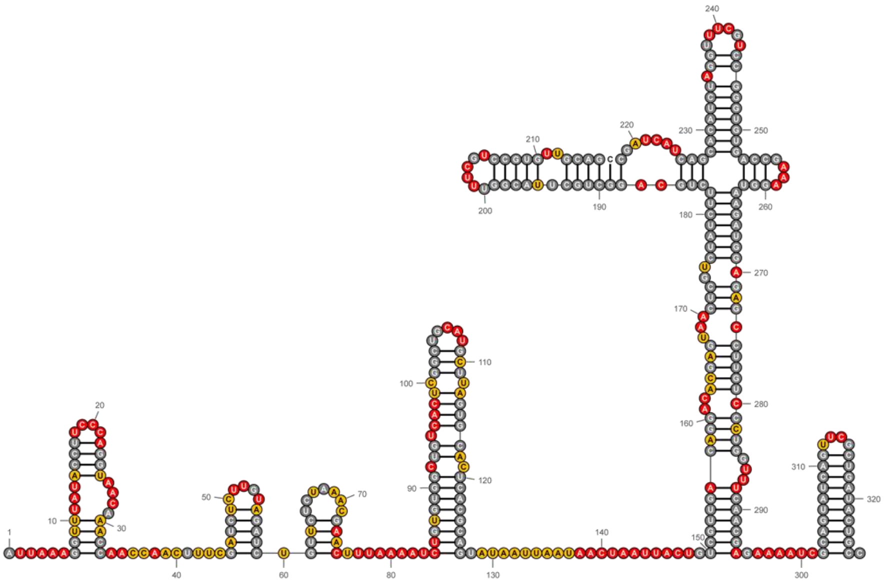

Figure S9, related to figure 8 *Secondary structure of SL 1-5 SARS CoV-2 5' UTR using replicate 1 DMS probing data at pH 8. Nucleotides are colored according to reactivities normalized by ShapeMapper2.2 with the "--dms" flag such that reactivities of 0-0.15 are gray, 0.15-0.4 are orange, and >0.4 are red.*

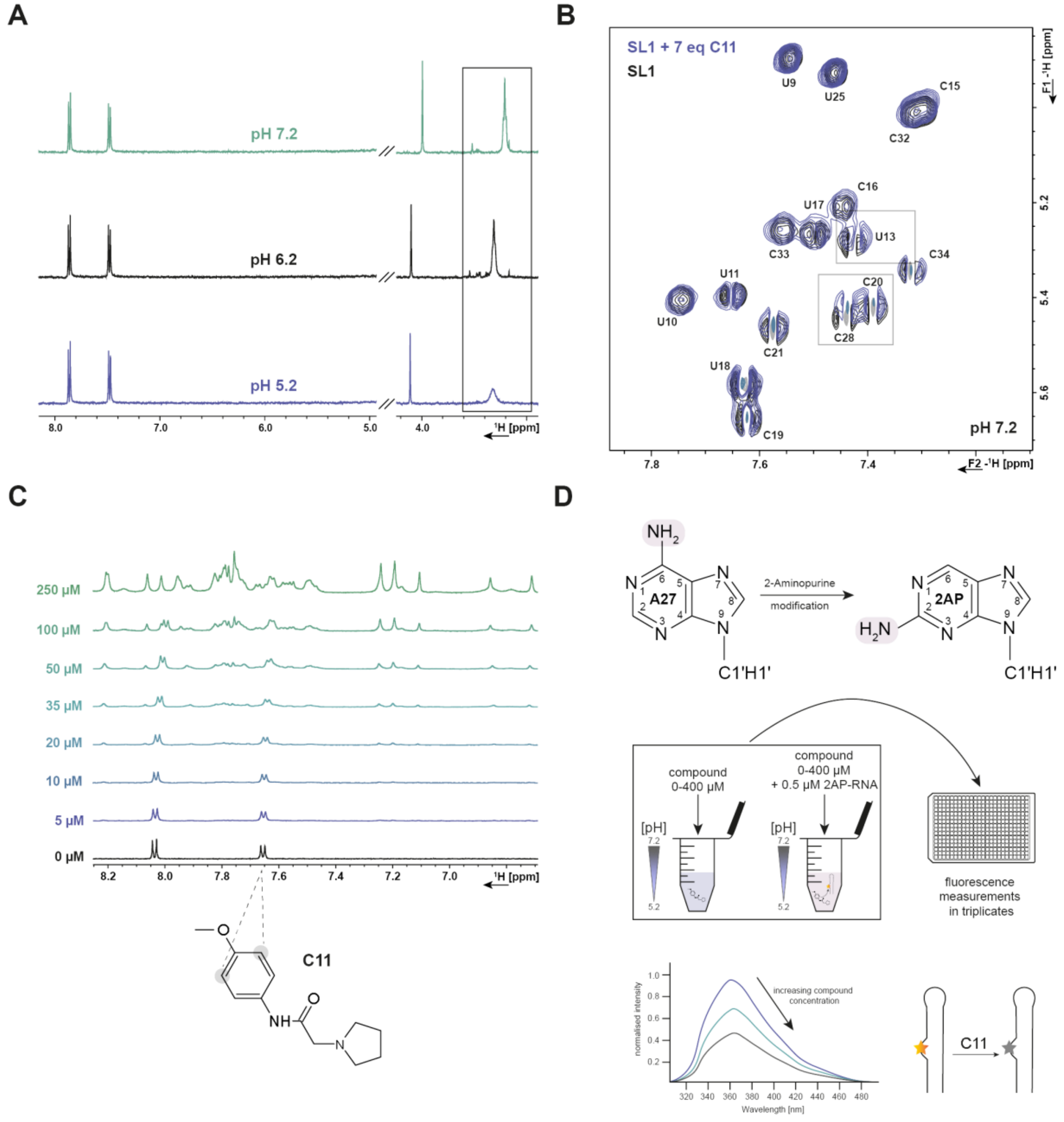

Figure S10, related to figure 9 *Targeting of SARS-CoV-2 SL1.* ***A*** *1D-^{1}H spectra of C11 at different pH values highlighting the pH sensitive resonances of C11. NMR spectra were acquired at 500 MHz and 298 K.* ***B*** *2D-^{1}H,^{1}H-TOCSY overlay of SL1 alone and SL1 after C11 addition reveals CSPs mapped onto the secondary structure of SL1. C11 was added in excess with an [RNA]:[ligand]-ratio of 1:7 (150 µM:1 mM). NMR spectra were acquired at 600 MHz and 298 K.* ***C*** *1D-^{1}H NMR spectra of the ligand-based titrations with C11 and SL1 at pH 7.2. NMR spectra were acquired at 600 MHz and 298 K with 100 µM C11 and 0-250 µM SL1.* ***A+C*** *Spectra were calibrated on DMSO-d_6 (2.50 ppm) and* ***B*** *on DSS (0.00 ppm); all samples were measured in 25 mM KPi (pH 5.2-7.2), 50 mM KCl and 5% DMSO-d_6.* ***D*** *2-Aminopurine modification of SL1 A27 and the assay set-up. Increasing compound concentration induces a decrease of the intrinsic 2AP fluorescence due to binding of the labelled RNA (highlighted by the greyed star).*

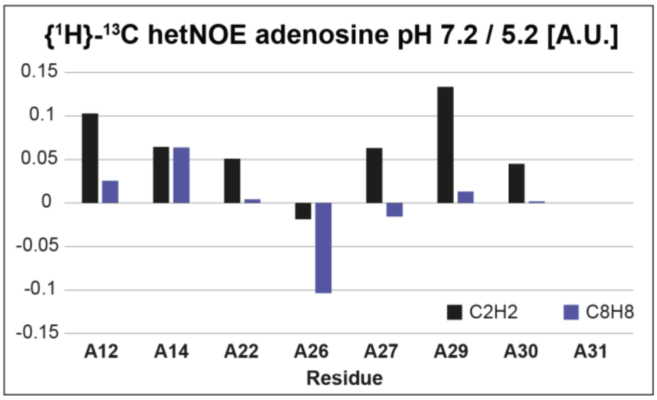

***Figure S11** hetNOE ratios for the aromatic resonances C2H2 (black) and C8H8 (blue) of adenosines of SL1 measured at 298 K and pH 7.2 and pH 5.2 plotted against the respective residues. The hetNOE ratio for A31, which is located most distant from any observed protonation event, was set to 1 and deviations from 1 are given. Values above 0 correspond to greater flexibility at pH 7.2 compared to 5.2, whereas ratios blow 0 correspond to greater flexibility at pH 5.2 versus pH 7.2. Experiments were recorded at 800 MHz with a selectively ^{13}C A/C labeled sample.*

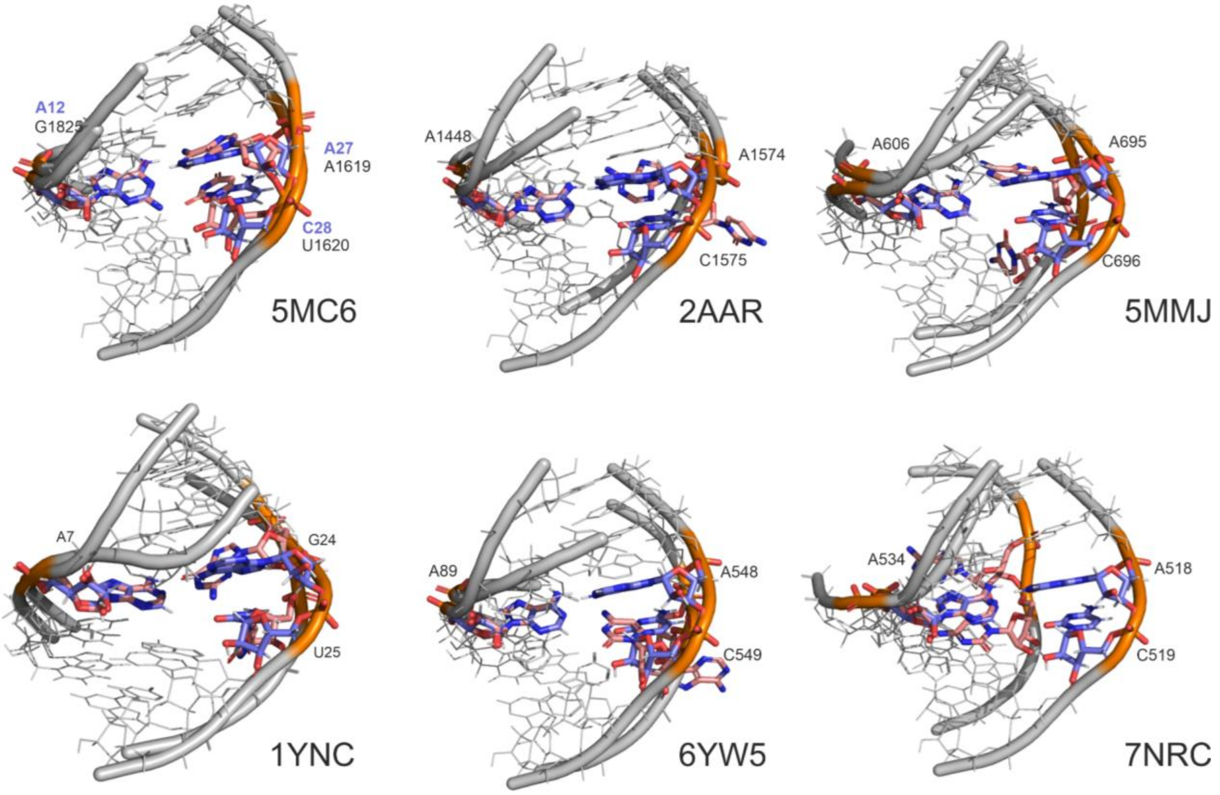

***Figure S12** Motif search; comparison of lowest-energy NMR structure of SL1 (blue sticks) with a set of different internal 1:2 loops form an RNA secondary structure motif search (*https://www.rnacossmos.com/search.php) (8)*. Salmon sticks) showing the preferential arrangement of A12 and C28 isosteric to a G•U wobble base pair as present in the 1:2 internal loop of 5MC6. The sequentially identical internal loops (A:AC; present in 2AAr, 5MMJ, 6YW5, 7NRC) show greater structure*

devitions to the SL1 conformation than the G:AU loop of 5MC6. The pdb structure 1YNC has a single cytidine 3'-bulge but shows a similar arrangement o the three bases A7, C24, and U25 to SL1's A12, A27 and C28.

Table S1, related to figure 2 *Comparison of R_g and D_{max} from prediction for NMR ensemble and SAXS data analysis. Hydrodynamic properties were calculated using the HullRad online server (http://52.14.70.9/)* (9).

	**NMR ensemble average structure**	**lowest-energy NMR structure**	**SAXS analysis**
**R_g (Å)**	16.74	16.63	17.3
**D_{max} (Å)**	59.34	56.08	56.4
**MW (kDa)**	9.46 (as 5'-triphosphate)		10.4; cred. int.= 9.05-10.85

Table S2, related to figure 3C *RDC values sorted by residue number (colunm 1) and bond vector (column 2) used for structure calculation (colunm 3) and structure validation (column 4).Validation results are shown in* ***Figure 3C*** *of the main text.*

**residue**	**bond vector**	**RDC [Hz]**<br><br>**ARIA calculation input**	**RDC [Hz]**<br><br>**PALES fitting input**
G7	N1H1	*not used*	-0.19 ± 2.4
G8	N1H1	*not used*	8.7 ± 1.2
U9	N3H3	*not used*	5.44 ± 1.2
U10	N3H3	*not used*	3.85 ± 1.2
G23	N1H1	*not used*	9.59 ± 1.2
G24	N1H1	*not used*	4.91 ± 1.2
U25	N3H3	*not used*	6.62 ± 1.2
A12	C8H8	10.01 ± 1.5	10.01 ± 2.5
A14	C8H8	13.9 ± 1.5	13.9 ± 2.5
A22	C2H2	12.1 ± 1.5	12.1 ± 2.5
A22	C8H8	12.21 ± 1.5	12.21 ± 2.5
A26	C2H2	12.96 ± 1.5	12.96 ± 2.5
A27	C2H2	8.18 ± 1.5	8.18 ± 2.5
A29	C2H2	15.11 ± 1.5	15.11 ± 2.5
A29	C8H8	21.5 ± 1.5	21.5 ± 2.5
A30	C2H2	18.9 ± 1.5	18.9 ± 4.5
A30	C8H8	20.6 ± 1.5	20.6 ± 2.5
A31	C2H2	12.8 ± 1.5	12.8 ± 2.5

Table S3 *4-Y-Loops in the pdb database (*http://rna.bgsu.edu/webfr3d/Results/64ab18ee6e44b/64ab18ee6e44b.html)(10). The search was performed to find potential preferred arrangements of 4Y-tetraloops (positions 2-5), closed by a Y:R base-pair (positions 1 and 6). No such preferential conformation, however, was observed for the 12 structured listed below.

**#**	**Reso-lution**	**Position 1**	**Position 2**	**Position 3**	**Position 4**	**Position 5**	**Position 6**	**Sequence**
1	3.2	6SWD|1|2|C|84	6SWD|1|2|U|85	6SWD|1|2|U|86	6SWD|1|2|C|87	6SWD|1|2|U|88	6SWD|1|2|G|89	C-U-U-C-U-G
2	2.25	7ZHG|1|2|C|84	7ZHG|1|2|U|85	7ZHG|1|2|U|86	7ZHG|1|2|C|87	7ZHG|1|2|U|88	7ZHG|1|2|G|89	C-U-U-C-U-G
3	6.6	4V6U|1|A2|C|72	4V6U|1|A2|U|73	4V6U|1|A2|U|74	4V6U|1|A2|C|75	4V6U|1|A2|U|76	4V6U|1|A2|G|77	C-U-U-C-U-G
4	9	4V4N|1|B2|C|72	4V4N|1|B2|U|73	4V4N|1|B2|U|74	4V4N|1|B2|C|75	4V4N|1|B2|U|76	4V4N|1|B2|G|77	C-U-U-C-U-G
5	2.35	7QIW|1|2|C|773	7QIW|1|2|U|774	7QIW|1|2|U|775	7QIW|1|2|C|776	7QIW|1|2|U|777	7QIW|1|2|G|778	C-U-U-C-U-G
6	5.5	4V7E|1|Aa|C|766	4V7E|1|Aa|U|767	4V7E|1|Aa|U|768	4V7E|1|Aa|C|769	4V7E|1|Aa|U|770	4V7E|1|Aa|G|771	C-U-U-C-U-G
7	2.4	7P7Q|1|a|U|867	7P7Q|1|a|U|868	7P7Q|1|a|U|869	7P7Q|1|a|C|870	7P7Q|1|a|C|871	7P7Q|1|a|G|872	U-U-U-C-C-G
8	2.9	7NHN|1|a|U|851	7NHN|1|a|U|852	7NHN|1|a|U|853	7NHN|1|a|C|854	7NHN|1|a|C|855	7NHN|1|a|G|856	U-U-U-C-C-G
9	9	4V4N|1|A1|C|2816	4V4N|1|A1|U|2817	4V4N|1|A1|C|2818	4V4N|1|A1|C|2819	4V4N|1|A1|C|2820	4V4N|1|A1|G|2821	C-U-C-C-C-G
10	6.6	4V6U|1|B1|C|2816	4V6U|1|B1|U|2817	4V6U|1|B1|C|2818	4V6U|1|B1|C|2819	4V6U|1|B1|C|2820	4V6U|1|B1|G|2821	C-U-C-C-C-G
11	2.98	7R6Q|1|1|C|1597	7R6Q|1|1|U|1606	7R6Q|1|1|U|1607	7R6Q|1|1|C|1608	7R6Q|1|1|C|1609	7R6Q|1|1|G|1610	C-U-U-C-C-G
12	2.5	5SWD|1|B|U|47	5SWD|1|B|U|48	5SWD|1|B|U|49	5SWD|1|B|C|50	5SWD|1|B|U|51	5SWD|1|B|A|52	U-U-U-C-U-A

Table S4, related to figure 5 *The distribution of distances of the A^{+}•C wobble base pair between A12N6 and C28O3, A12N6 and C28O2, and A12N1 and C26O2, respectively. The distances representing a perfect A^{+}•C-wobble geometry are shown for comparison.*

	**A12N6 to C28N3 [Å]**	**A12N1 to C28O2 [Å]**	**A12N6 to C28O2 [Å]**
SL1 state 1	6.3	5	5.1
SL1 state2	5.5	4.2	4.7
SL1 state3	6.4	4.5	5.4
SL1 state4	4.9	3.8	4.7
SL1 state5	5.1	3.5	5.3
SL1 state6	5.2	4.6	3.6
SL1 state7	4.8	3.7	3.8
SL1 state8	7.4	4.9	6.8
SL1 state9	5.8	5.1	3.9
SL1 state10	6	4.1	5.1
average SL1	5.7 ± 0.88	4.3 ± 0.6	4.8 ± 0.9
402D state 1	3.5	3.1	3.3
402D state 2	3.1	2.8	2.9
average 402D	3.3	2.95	3.1

Table S5, related to figure 8 *Template and primer sequences used in PD-MaP experiments. Underlined portion of template denotes T7 RNA Polymerase promotor region.*

**Name**	**Sequence**
SARS CoV-2 5' UTR Template	CTAATACGACTCACTATAGGGCCTTCGGGCCAAATTAAAGGTT<br>TATACCTTCCCAGGTAACAAACCAACCAACTTTCGATCTCTTGT<br>AGATCTGTTCTCTAAACGAACTTTAAAATCTGTGTGGCTGTCA<br>CTCGGCTGCATGCTTAGTGCACTCACGCAGTATAATTAATAAC<br>TAATTACTGTCGTTGACAGGACACGAGTAACTCGTCTATCTTC<br>TGCAGGCTGCTTACGGTTTCGTCCGTGTTGCAGCCGATCATC<br>AGCACATCTAGGTTTCGTCCGGGTGTGACCGAAAGGTAAGAT<br>GGAGAGCCTTGTCCCTGGTTTCAACGAGAAAATCGCAGTATC<br>AGTTCGCTGATACTGCCAAATCGGGCTTCGGTCCGGTTC
5' Template Primer	CTAATACGACTCACTATAGGGCCTTCGGG
3' Template Primer	GAACCGGACCGAAGCCCG
5' Primer (Step 1)	CCCTACACGACGCTCTTCCGATCTNNNNNGGCCTTCGGGCC<br>AA
3' Primer (Step 2)/ RT Primer	GACTGGAGTTCAGACGTGTGCTCTTCCGATCTNNNNNTTGAA<br>CCGGACCGAA

Table S6, related to figure 8 *Read depths per probing condition per replicate.*

**Condition**	**Replicate 1**	**Replicate 2**	**Replicate 3**
**pH 8**	772.076	603.442	741.844
**pH 5**	627.096	597.429	495.674
**Control**	582.481	544.193	657.652

REFERENCES

1. Richter,C., Hohmann,K.F., Toews,S., Mathieu,D., Altincekic,N., Bains,J.K., Binas,O., Ceylan,B., Duchardt-Ferner,E., Ferner,J., *et al.* (2021) 1H, 13C and 15N assignment of stem-loop SL1 from the 5'-UTR of SARS-CoV-2. *Biomol. NMR Assign.*, **15**, 467–474.

2. Mori,S., Abeygunawardana,C., Johnson,M.O. and Van Zljl,P.C.M. (1995) Improved sensitivity of HSQC spectra of exchanging protons at short interscan delays using a new fast HSQC (FHSQC) detection scheme that avoids water saturation. *J. Magn. Reson. B*, **108**, 94–98.

3. Shaka,A.J., Lee,C.J. and Pines,A. (1988) Iterative schemes for bilinear operators; application to spin decoupling. *J. Magn. Reson.*, **77**, 274–293.

4. Hwang,T.L. and Shaka,A.J. (1995) Water Suppression That Works. Excitation Sculpting Using Arbitrary Wave-Forms and Pulsed-Field Gradients. *J. Magn. Reson. Ser. A*, **112**, 275–279.

5. Schanda,P. and Brutscher,B. (2005) Very fast two-dimensional NMR spectroscopy for real-time investigation of dynamic events in proteins on the time scale of seconds. *J. Am. Chem. Soc.*, **127**, 8014–8015.

6. Skalicky,J.J., Sukumaran,D.K., Mills,J.L. and Szyperski,T. (2000) Toward structural biology in supercooled water [8]. *J. Am. Chem. Soc.*, **122**, 3230–3231.

7. Skalicky,J.J., Mills,J.L., Sharma,S. and Szyperski,T. (2001) Aromatic ring-flipping in supercooled water: implications for NMR-based structural biology of proteins. *J. Am. Chem. Soc.*, **123**, 388–397.

8. Richardson,K.E., Kirkpatrick,C.C. and Znosko,B.M. (2020) RNA CoSSMos 2.0: an improved searchable database of secondary structure motifs in RNA three-dimensional structures. *Database*, **2020**, 153.

9. Fleming,P.J. and Fleming,K.G. (2018) HullRad: Fast Calculations of Folded and Disordered Protein and Nucleic Acid Hydrodynamic Properties. *Biophys. J.*, **114**, 856–869.

10. Petrov,A.I., Zirbel,C.L. and Leontis,N.B. (2011) WebFR3D - A server for finding, aligning and analyzing recurrent RNA 3D motifs. *Nucleic Acids Res.*, **39**, W50–W55.
